# Induction of Meiosis from Human Pluripotent Stem Cells

**DOI:** 10.1101/2024.05.31.596483

**Published:** 2024-05-31

**Authors:** Merrick Pierson Smela, Jessica Adams, Carl Ma, Laura Breimann, Ursula Widocki, Toshi Shioda, George M. Church

**Affiliations:** 1 Wyss Institute, Harvard University; Boston, 02215, USA; 2 Department of Genetics, Harvard Medical School; Boston, 02115, USA; 3 Broad Institute of MIT and Harvard; Cambridge, 02138, USA; 4 Mass. General Research Institute; Boston, 02129, USA

## Abstract

An *in vitro* model of human meiosis would accelerate research into this important reproductive process and development of therapies for infertility. We have developed a method to induce meiosis starting from male or female human pluripotent stem cells. We demonstrate that DNMT1 inhibition, retinoid signaling activation, and overexpression of regulatory factors (anti-apoptotic BCL2, and pro-meiotic HOXB5, BOLL, or MEIOC) rapidly activates meiosis, with leptonema beginning at 6 days, zygonema at 9 days, and pachynema at 12 days. Immunofluorescence microscopy shows key aspects of meiosis, including chromosome synapsis and sex body formation. The meiotic cells express genes similar to meiotic oogonia *in vivo*, including all synaptonemal complex components and machinery for meiotic recombination. These findings establish an accessible system for inducing human meiosis *in vitro*.

All sexually reproducing species rely on meiosis to produce haploid gametes from diploid germ cells. To date, the most detailed studies of meiosis have taken place in non-human organisms, due to the lack of a reliable *in vitro* model of human meiosis, as well as technical and ethical barriers to obtaining meiotic cells from humans. Therefore, a method of inducing meiosis in cultured human cells could greatly advance the study of this crucial reproductive process, and could also lead to new therapies for people with infertility.

Research on animals such as mice has revealed important characteristics of mammalian meiosis, including requirements of erasing DNA methylation (1), as well as retinoic acid and BMP signaling from gonadal somatic cells (2). Recent studies have demonstrated the initiation of meiosis in mouse cells *in vitro* (2–8), even producing viable offspring from the resulting gametes (3, 4). However, studies attempting to initiate meiosis in human cells have been less successful. These studies based their main conclusions on the production of haploid (1N 1C) cells as assessed by flow cytometry for DNA content (9–12). However, this approach has two important flaws (13). First, the 1N 1C state is non-physiological in eggs since meiosis is not completed until after fertilization. Second, dead and dying cells with fragmented nuclei can have reduced DNA content, leading to false positives in this assay. Some studies also examined the expression of the meiotic markers SYCP3 and γH2AX (11, 12, 14, 15), but did not convincingly show the expected localization of these proteins during the stages of meiosis, and our attempts to reproduce these protocols were unsuccessful (Table S1).

Here, we present an *in vitro* model of meiosis from human induced pluripotent stem cells (hiPSCs). By screening conditions for activating the expression of meiotic genes, we found that DNMT1 inhibition, retinoic acid receptor activation, and overexpression of anti-apoptosis and pro-meiosis factors can rapidly initiate meiosis in male and female hiPSCs. We show that this method generates cells corresponding to the leptotene, zygotene, and pachytene stages of meiosis, and that these cells have gene expression similar to meiotic germ cells *in vivo*. Overall, our method will be a useful tool for researchers studying human meiosis, and with further optimization may allow the production of human gametes *in vitro*.

## Barcode enrichment screening of candidate meiosis-promoting factors

**Fig. 1.**
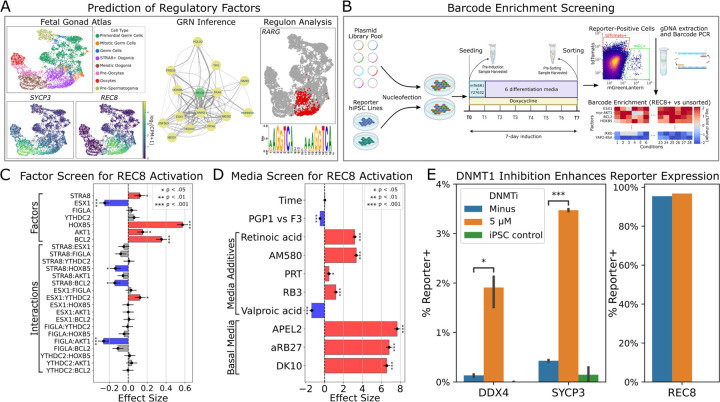
Screening of regulatory factors for reporter activation. (A) Prediction of regulatory factors based on fetal gonad scRNAseq data. (B) Barcode enrichment screening in reporter iPSCs (see Methods). (C) Fractional factorial screen (32 combinations, each tested in 2 cell lines) of seven top factors for REC8 activation. Effect sizes and significance were calculated using a linear model on logit-transformed data. (D) Screen of media and additives for REC8 activation (see Methods). Effect sizes and significance were calculated as above. (E) Effects of DNMT1i treatment on expression of DDX4, SYCP3, and REC8 reporters (n=4).

We began by analyzing previously published scRNAseq data of human fetal gonads, which contain a variety of cell types (16), including pre-meiotic STRA8+ oogonia and fully meiotic oogonia/oocytes (Fig. 1A). We confirmed *REC8* as a reliable marker for early meiotic cells, and *SYCP3* for late meiotic cells. Using previously constructed male and female DDX4-tdTomato reporter hiPSCs (17), we engineered dual reporter lines for DDX4-tdTomato/REC8-mGreenLantern (D4TR8G) and DDX4-tdTomato/SYCP3-mGreenLantern (D4TS3G) (Fig. S1). We validated these lines by whole genome sequencing (18), and by CRISPRa and flow cytometry.

Next, we chose 78 candidate meiosis-promoting factors based on scRNAseq analysis and previous literature (see Methods). We cloned these into a barcoded PiggyBac transposon plasmid for doxycycline-inducible expression. We integrated the library into the reporter hiPSCs, activated expression, and sorted reporter-positive cells after seven days of induction (Fig. 1B). We tested low-copy and high-copy integration, as well as a variety of different culture media (see Methods). Comparing barcode frequencies between reporter-positive and unsorted populations, we found several factors consistently enriched in REC8+ cells (Fig. S2), although results for the other reporters were noisy due to low cell yield.

### Optimization of REC8 activation

Based on the barcode enrichment results, we narrowed down our library to sixteen factors and tested these individually for activation of REC8 and SYCP3 expression (Fig. S3). BCL2, HOXB5, and myr-AKT1 all slightly activated REC8, although no factors activated SYCP3. We next performed a combinatorial screen of seven factors, and found that BCL2, HOXB5, STRA8, and myr-AKT1 all significantly promoted REC8 expression (Fig. 1C). Using these top four factors, we tested different media compositions, supplements, and induction times (Fig. 1D). The best-performing medium was APEL2, and retinoids (retinoic acid and AM580) significantly increased REC8 expression. The PRC1 inhibitors RB3 and PRT4164 caused a small increase in REC8 expression, but this was associated with extensive toxicity. Valproic acid was also toxic, and significantly decreased REC8 expression. There was no significant change in REC8 expression between 6, 7, and 8 days of induction.

### DNMT1 inhibition upregulates meiotic markers

Using the top factors and differentiation medium, we could induce REC8+ cells at nearly 100% efficiency, but cells still lacked SYCP3 expression. We reasoned that overexpressing meiosis-promoting factors might not be sufficient, and that downregulation of meiosis-inhibiting factors might be required. Therefore, we tested CRISPRi knockdown of ten epigenetic factors. Knockdown of *DNMT1* resulted in a small upregulation of SYCP3 (Fig. S4). In previous work, we used a noncovalent DNMT1 inhibitor, GSK3484862, to erase DNA methylation and establish an oogonia-like epigenetic state (17). Treatment with this inhibitor resulted in a significant increase in expression of SYCP3, as well as DDX4 (Fig. 1E).

### scRNAseq screening identifies meiotic cells and associated factors

**Fig. 2.**
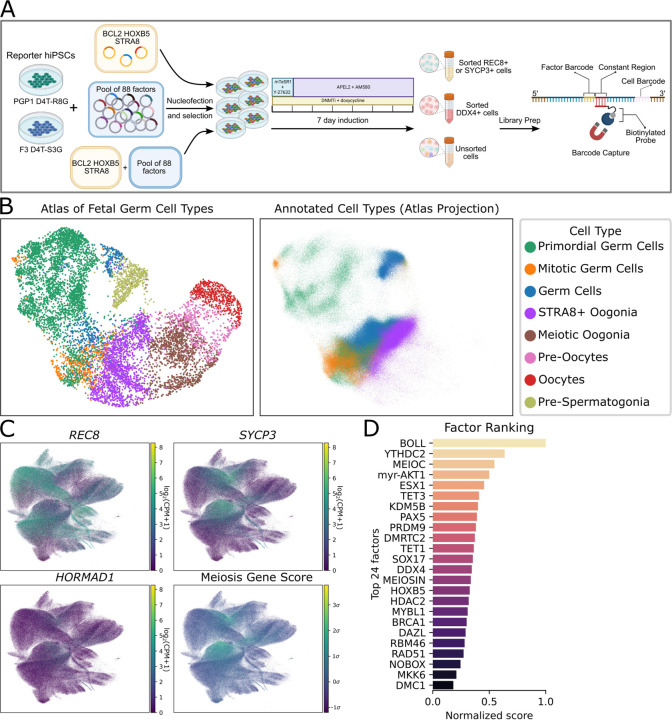
scRNAseq identifies factors promoting meiotic cell identity. (A) scRNAseq differentiation, library prep, and barcode capture (see Methods). (B) Cell type annotation based on the fetal germ cell atlas. (C) Expression of selected meiosis marker genes, as well as the meiosis gene score calculated from expression of 19 meiosis-specific genes. (D) Factor ranking based on barcode overrepresentation in cells with meiotic gene expression.

With DNMT1 inhibition, retinoid treatment, and overexpression of BCL2, HOXB5, and STRA8, we observed activation of REC8, SYCP3, and DDX4. However, we wanted to take a broader view of the gene expression in our cells, and see if expressing any additional factors could drive the cells closer to meiosis. Therefore, we generated iPSC populations containing integrated expression vectors for BCL2, HOXB5, and STRA8 under hygromycin selection, as well as a pool of 88 other candidate regulatory factors under puromycin selection (Supplementary Table 2). Following our induction protocol, we performed scRNAseq on sorted reporter-positive cells as well as unsorted cells (Fig. 2A, and see Methods).

We first investigated whether any meiotic cells were present in our samples. Leveraging the fetal gonad scRNAseq dataset (16), we performed cell type annotation (Fig. 2B). The majority of our cells were classified as pre-meiotic oogonia, and small fraction of cells were annotated as fully meiotic. The proportion of these cells was greatest (0.8%) in sorted SYCP3+ samples. As another way of looking for meiotic cells, we constructed a score based on expression of meiosis-specific genes. In addition to *REC8* and *SYCP3*, the markers for which we sorted, we also observed expression of other essential meiosis genes, such as *HORMAD1*, in a smaller fraction of cells (Fig. 2C). We chose a list of nineteen meiosis-specific genes (Supplementary Table 3) and scored cells based on their expression. Out of 646,493 cells in our dataset, 1,276 cells had a gene score >4σ, compared with an expected 20 cells assuming random gene expression.

Next, we asked which regulatory factors were responsible for generating these meiotic cells. We performed a hybridization-based capture to enrich our scRNAseq library for barcode sequences and identified at least one expressed barcode in 91% of our cells. We then examined which factors were overrepresented in meiotic cells (defined using cell type annotation or gene scoring) versus pre-meiotic cells. We chose the top 24 for subsequent screening (Fig. 2D).

### Optimization of factors for inducing meiosis

We next expressed each of these 24 factors along with the previous top three (BCL2, HOXB5, and STRA8). After seven days of induction, we monitored reporter activation and performed immuno-staining for the meiosis markers HORMAD1 and SYCP3. We identified four factors, BOLL, MEIOC, MEIOSIN, and myr-AKT1, as the most promising (Fig. S5A). We combined these four with the previous top three and repeated the experiment, this time analyzing a series of time points (7, 9, 13, and 16 days). Excitingly, at days 9 and 13 post-induction, we observed a few HORMAD1+ SYCP3+ cells with zygotene-like filamentous staining (Fig. S5B).

In order to narrow down which factors were responsible for inducing meiosis, we performed two rounds of combinatorial screening. In the first round, we tested 16 combinations of the initial set of seven factors (Fig. S5CDE). No combination lacking BCL2 was able to induce zygotene cells as measured by HORMAD1 filament formation. BCL2/HOXB5/BOLL and BCL2/HOXB5/myr-AKT1/MEIOC were the best individual combinations. BCL2, HOXB5, and BOLL all induced a significant increase in the number of zygotene cells. Interestingly, we found that STRA8 significantly decreased the zygotene score when overexpressed.

**Fig. 3.**
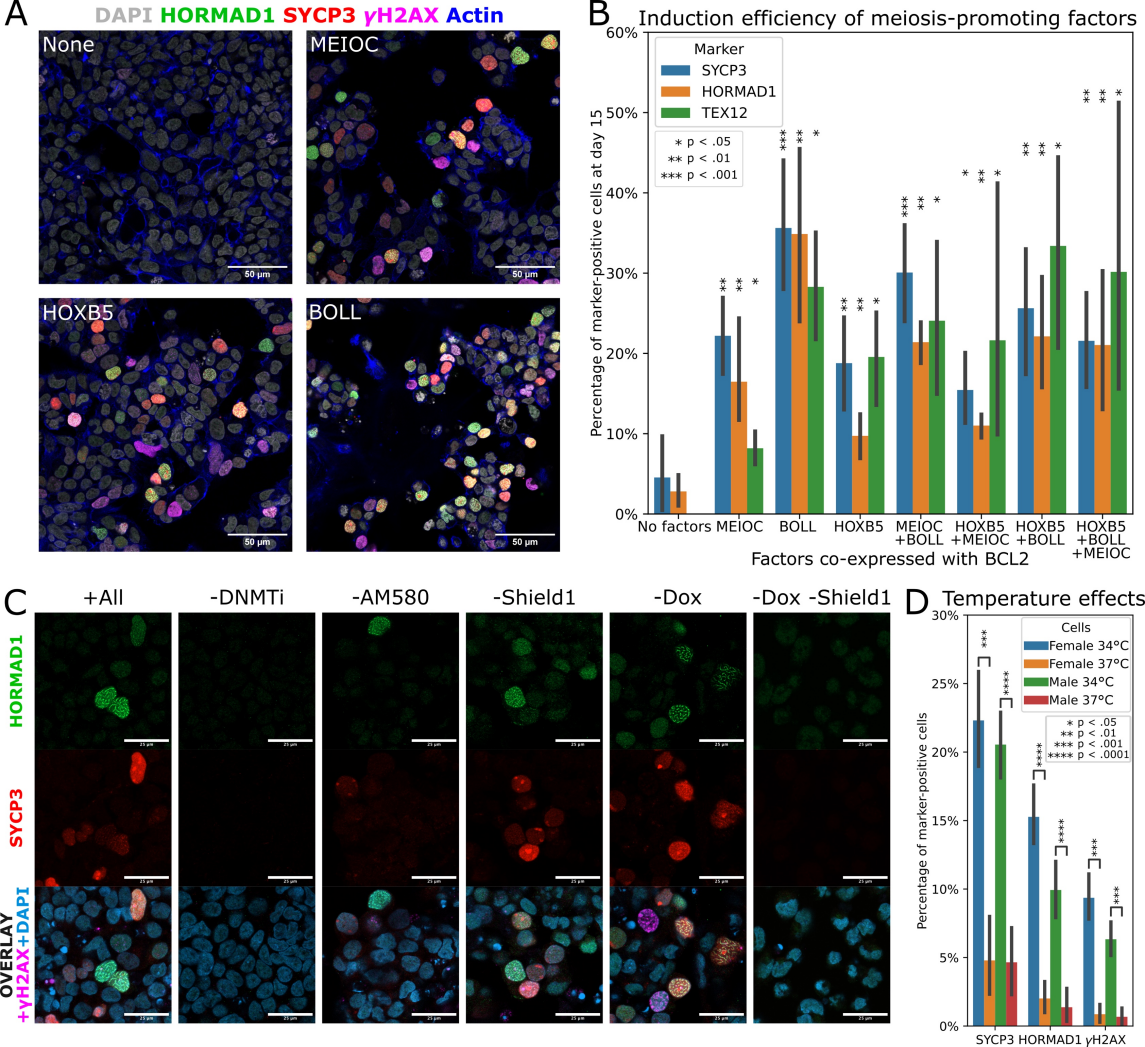
Optimization of meiosis induction. (A) Cells expressing constitutive BCL2 and Dox-inducible MEIOC, HOXB5, or BOLL were subjected to the meiosis induction protocol (see Methods) and stained for DNA (DAPI), actin (phalloidin), HORMAD1, SYCP3, and γH2AX (scale bar 50 µm). (B) From the same experiment as panel A, all eight possible combinations of the factors tested in three cell lines. Two images were analyzed per cell line and combination. (C) Cells expressing constitutive BCL2, Dox-inducible BOLL, and Shield1-inducible HOXB5 were subjected to the meiosis induction protocol omitting various factors (see Methods). Representative immunofluorescence images are shown (scale bar 25 µm). (D) Effects of performing meiosis induction in male and female hiPSCs at 34 °C or 37 °C (n = 4 samples per condition).

Finally, we generated hiPSCs constitutively expressing BCL2, and inducibly expressing HOXB5, BOLL, and MEIOC, testing all eight possible combinations of these three factors. We observed that each of the factors could induce meiosis when expressed with BCL2 (Fig. 3A). BOLL was the most efficient of the three factors we tested (Fig. 3B). In the BCL2-only control, we observed no HORMAD1+ cells and only a few occasional SYCP3+ cells.

### DNA demethylation and retinoid stimulation are required for efficient meiotic initiation

To further investigate the conditions necessary for meiotic initiation, we omitted different components of our protocol (Fig. 3C, Fig. S6). Without DNMT1 inhibition, meiosis was completely blocked. However, DNMT1 inhibitor could be withdrawn after the first five days without negatively affecting results. Omitting the retinoid AM580 resulted in fewer meiotic cells, but some were still present. If AM580 treatment was started later than day five, results were similarly poor. Finally, using orthogonal induction systems (Dox and Shield1) for BOLL and HOXB5 expression, we confirmed that expression of at least one of these factors was required for inducing meiosis.

### Lower temperatures enhance meiotic induction

In males, meiosis takes place in the adult testes, which are cooler than the rest of the body. Previous studies indicated that male meiosis is less efficient at 37 °C (8). Therefore, we tested our meiosis induction protocol at 34 °C vs. 37 °C using male and female hiPSCs. Meiosis induction was significantly enhanced at 34 °C in not only male, but also female cells (Fig. 3D, Fig. S7A). We tested 34 °C starting at either day 1 or day 3 of the induction protocol, and found that both worked equally well. Monitoring the cells for up to 21 days, we saw that cell viability declined past day 16. Interestingly, we noticed that REC8-mGreenLantern fluorescence was much weaker at 34 °C compared to 37 °C, whereas SYCP3-mGreenLantern and DAZL-mGreenLantern were equally bright (Fig. S7B).

### Identification of stages of meiosis

**Fig. 4.**
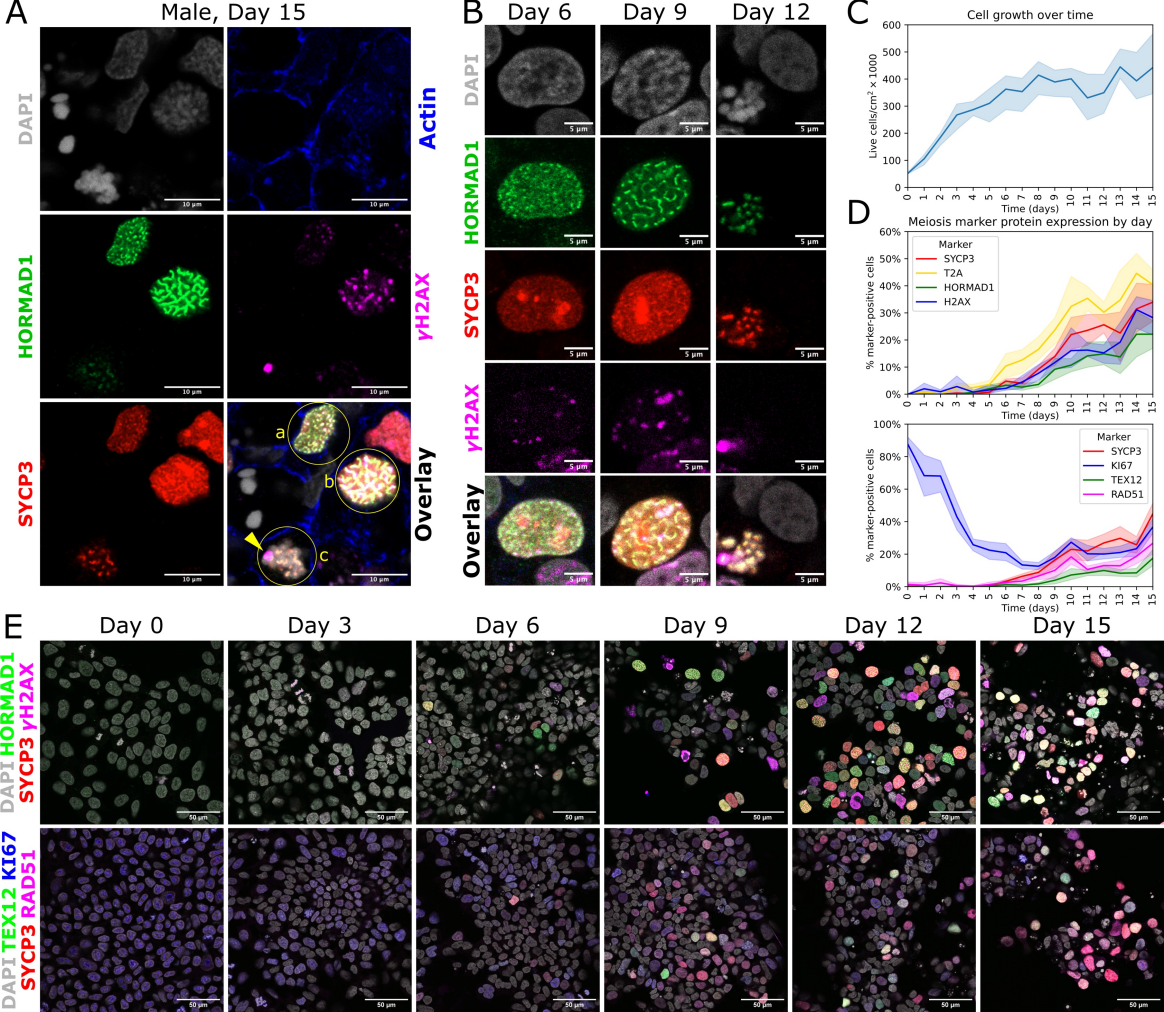
Progression of stages of meiosis over time. (A) Immuno-staining of day 15 male meiotic cells. Three stages of meiosis are visible: (a) leptonema, (b) zygonema, and (c) pachynema. A γH2AX-positive sex body, labeled with an arrow, is visible on the periphery of the pachytene nucleus. Scale bar is 10 µm. (B) Representative images of nuclei at different time points during meiosis induction in male hiPSCs. Scale bar is 5 µm. (C) Cell growth over 15 days of meiosis induction (two female and one male hiPSC line). (D) Meiosis marker protein expression over 15 days of induction (two female and one male hiPSC line, two images analyzed per line per time point). REC8 expression was measured by staining for the T2A linker peptide. (E) Representative images of meiosis marker expression over time. Scale bar is 50 µm.

To analyze which stages of meiosis were present in our cells, we performed co-staining for HORMAD1, which marks the chromosome axis and is removed from synapsed chromosomes during pachynema, SYCP3, which marks the lateral elements of the synaptonemal complex, and γH2AX, which marks recombination-related DNA damage in leptonema and zygonema, and the sex body (unsynapsed XY chromosomes) of male cells in pachynema and diplonema (19). By day 12 of our induction protocol, three stages of meiosis (leptonema, zygonema, and pachynema) were visible. A representative image of these three stages is shown in Fig. 4A. The leptotene cell (labeled a) has diffuse HORMAD1 and SYCP3 expression and a faint γH2AX signal. The zygotene cell (labeled b) has filamentous HORMAD1 and SYCP3, and stronger γH2AX. Additionally, the chromosomes are starting to compact, as seen in the DAPI channel. The pachytene cell (labeled c) has fully compacted chromosomes, associated with SYCP3 staining. HORMAD1 staining is much weaker, and a γH2AX positive sex body (labeled with an arrow) is visible on the nuclear periphery. A 3D z-stack of this image is provided as Supplementary Video 1. Meiotic cells also expressed cytoplasmic DDX4, and nuclear foci of the recombination marker RAD51 (Fig. S8).

### Meiotic progression over 15 days of induction

Using our optimized protocol, we investigated the progression of meiosis over time. Using three hiPSC lines (two female and one male), we measured a total of 16 timepoints per line (Fig. 4 and Supplementary Data), every 24 hours from the beginning of our induction protocol (day 0; hiPSCs) through day 15. Leptotene cells were first seen at day 6, zygotene cells were first seen at day 9, and pachytene cells were first seen at day 12 (Fig. 4B). We counted the number of live cells at each timepoint. The cells proliferated ~8-fold over the first week of the protocol, but the number remained stable after day 8 (Fig. 4C). At the beginning of the protocol, nearly all cells were positive for KI67, a marker of proliferating cells as well as meiotic cells (20). As cell proliferation slowed, KI67 decreased from days 0–7, but remained expressed in meiotic cells (Fig. 4D).

REC8-T2A was the first meiotic marker to be expressed, starting at day 6 and continuously increasing through day 11 (Fig. 4D and E). HORMAD1, SYCP3, γH2AX, and RAD51 expression followed similar trajectories, starting around days 7–8 and increasing through day 15. TEX12, which is required for full chromosome synapsis in zygonema and pachynema (21), was the last marker to be expressed, starting around day 9 and increasing thereafter (Fig. 4D and E). The kinetics were similar in male and female hiPSC lines.

### Gene expression dynamics during meiosis induction

**Fig. 5.**
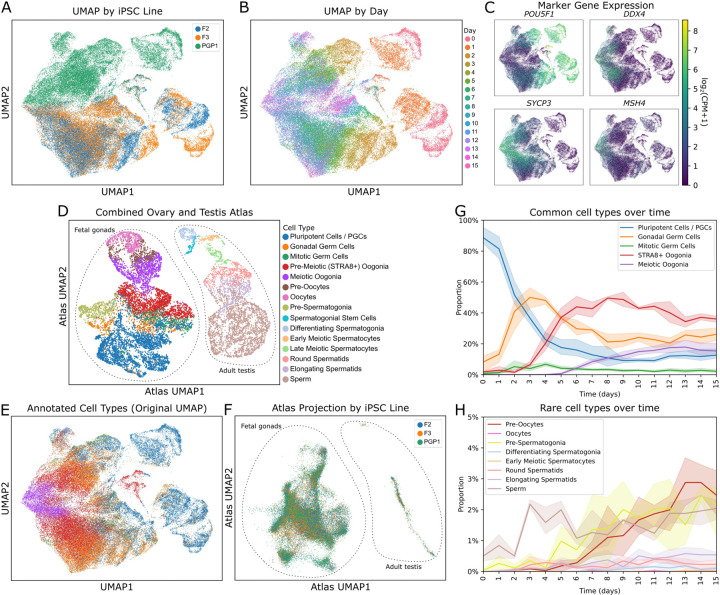
Timecourse scRNAseq analysis of meiosis induction. (A) UMAP plot, colored by hiPSC line. PGP1 is male; F2 and F3 are female. (B) UMAP plot, colored by day of sample collection. (C) UMAP plots colored by expression of marker genes for pluripotency (*POU5F1*), oogonia/gonocytes (*DDX4*), meiosis (*SYCP3*), and late-stage meiotic recombination (*MSH4*). (D) Cell types present in the combined ovary and testis reference atlas. (E) UMAP plot of annotated cell types over 15 days of meiosis induction. (F) iPSC-derived cells projected onto the atlas UMAP, colored by cell line. (G) Proportions of common (>5% abundance) cell types over time. (H) Proportions of rare (<5% abundance) cell types over time.

We next analyzed scRNAseq data from each day of our meiosis induction protocol. Our post-filtering dataset comprised a total of 69,018 cells from one male (PGP1) and two female (F2 and F3) cell lines, and sixteen time points spanning days 0 to 15. We first performed dimensionality reduction (Fig. 5A and 5B) and examined marker gene expression (Fig. 5C, Fig. S9). Cells from the two female lines overlapped, but the male cells were largely separate (Fig. 5A). However, at later time points, the male and female lineages converged (Fig. 5B). Expression of the pluripotency marker *POU5F1* was initially high (Fig. 5C), but quickly declined and reached low levels by day 6. At intermediate time points, cells began to express gonadal germ cell markers, including *DDX4* (Fig. 5C), *DAZL, MAEL, STK31,* and *MAGE* and *PIWI* family genes (Fig. S9). Cells also expressed marker genes for meiosis, including all components of the synaptonemal complex. As observed by immunofluorescence (Fig. 4), *REC8* was one of the earliest meiosis genes expressed. By days 12–15, a subset of cells expressed late-stage pachytene recombination markers such as *MSH4* (Fig. 5C, Fig. S9). Markers for gametes (oocytes and sperm) were not highly expressed.

To compare our cells with *in vivo* germ cells, we constructed a scRNAseq atlas by combining data from human fetal gonads (containing female meiotic cells) and adult testis (containing male meiotic cells) from two previously published atlases (Fig. 5D) (16, 22). We projected our cells onto the combined atlas and performed cell type annotation (Fig. 5E and 5F). This analysis showed that our cells were more similar to fetal ovarian cells, although a few cells were classified as adult testicular cells. When projected onto the atlas UMAP, cells from all three iPSC lines overlapped, and there was no clear distinction between male and female lines (Fig. 5F).

We next examined the proportions of each cell type over time, plotting common (>5% abundance) and rare (<5% abundance) cell types separately (Fig. 5G and 5H). Although the cells at early timepoints were largely annotated as primordial germ cells (PGCs), this reflects expression of marker genes such as *POU5F1* and *NANOG* which are shared between pluripotent cells and PGCs, rather than a *bona fide* PGC-like state. Indeed, our cells lacked expression of definitive PGC marker genes including *NANOS3*, *PRDM1*, *SOX17*, and *TFAP2C* (Fig. S8). Despite skipping over the PGC state, our cells transitioned through gonadal germ cell and oogonia-like states before entering meiosis (Fig. 5G). Fully meiotic cells were first present at day 6, with the proportion increasing through day 12. At later timepoints, some cells were classified as diplotene-arrested (pre-oocytes or oocytes) or post-meiotic (round spermatids, elongating spermatids, or sperm). The proportion of these cell types increased over time and reached a maximum on day 13 (Fig. 5H).

## Discussion

Here we report a reliable and rapid protocol for inducing meiosis in male and female human cells. Our method relies on overexpressing BCL2 and at least one meiosis-promoting factor. We identified HOXB5, BOLL, and MEIOC as able to perform this role. Of these, BOLL and MEIOC were previously reported as pro-meiotic (11, 23). HOXB5 was known to be expressed in fetal oogonia (16), but its role in meiosis was not previously studied. The most likely role of BCL2 in our protocol is to prevent apoptosis resulting from DNA double strand breaks during leptonema (24). However, it is possible that BCL2 plays an additional role, as BCL2 alone was sufficient to upregulate REC8 (Fig. S3B). In accordance with previous studies in mice (1, 2, 8), we additionally show that DNA demethylation is required for meiotic entry, and that retinoid treatment and lower temperatures increase the efficiency.

Comparing the gene expression of our cells to *in vivo* meiotic cells, we find meiotic cells induced from both male and female hiPSCs are more similar to meiotic oogonia *in vivo*, although a small fraction (<5%) are similar to meiotic spermatocytes. Although our cells express oogonia/gonocyte markers, they do not transition through a PGC-like stage prior to meiotic entry, suggesting that this stage is not required for meiosis.

The primary limitation of our method is its low efficiency in producing pachytene and later-stage cells. We are currently using polyclonal populations of iPSCs with randomly integrated expression vectors, and switching to a system that allows precise control of transgene expression levels may improve results. Furthermore, in cultured mouse spermatogonia, meiosis has lower efficiency and fidelity compared with meiosis *in vivo* (8), suggesting an important role for the gonadal niche. Thus, integrating meiotic cells into recently developed ovarian organoid systems may be beneficial (25, 26). Despite its modest efficiency, our current method is easily scalable and produces late-stage meiotic cells in a relatively short time (13–15 days), similar to the known duration of human meiosis (27).

The ability to induce meiosis using human cells *in vitro* will unlock new opportunities for science and medicine. Two examples include screening candidate male contraceptives, and using knockout hiPSCs to investigate effects of mutations. Future developments could allow the production of human gametes *in vitro*, or the generation of genetic crosses between different human cell lines. We believe that our method of inducing meiosis will greatly benefit research into this important reproductive process.

## Supplementary Material

Supplement 1

Supplement 2

## Data Availability

Sequencing data have been deposited to NCBI repositories under the accession GSE268385 [SRA accession pending]. Microscope images will be made available on Dryad following publication. Analysis code is available on Github: https://github.com/mpiersonsmela/meiosis Plasmids will be made available on Addgene following publication. All other materials, including cell lines, will be made available upon request under an MTA for noncommercial use.
