# Supplementary material for: Induction of Meiosis from Human Pluripotent Stem Cells
Source: bioRxiv. 2024 May 31:2024.05.31.596483. Preprint. [Version 1] doi: 10.1101/2024.05.31.596483 (PMC11160729; doi:10.1101/2024.05.31.596483)
Supplement: Supplement 2 [file NIHPP2024.05.31.596483v1-supplement-2.pdf]

bioRxiv preprint doi: https://doi.org/10.1101/2024.05.31.596483; this version posted June 1, 2024. The copyright holder for this preprint (which was not certified by peer review) is the author/funder, who has granted bioRxiv a license to display the preprint in perpetuity. It is made available under aCC-BY 4.0 International license.

Supplementary Materials:

Materials and Methods

Figs. S1 to S9

Tables S1 to S6

References (28–38)

Movie S1

bioRxiv preprint doi: https://doi.org/10.1101/2024.05.31.596483; this version posted June 1, 2024. The copyright holder for this preprint (which was not certified by peer review) is the author/funder, who has granted bioRxiv a license to display the preprint in perpetuity. It is made available under aCC-BY 4.0 International license.

Methods

iPSC culture

Human iPSC lines (ATCC-BXS0115, referred to as F2, ATCC-BXS0116, referred to as F3, and PGP1) were cultured in mTeSR Plus medium (Stemcell Technologies) on standard polystyrene plates coated with hESC-qualified Matrigel (Corning). hiPSCs were grown in a 37 °C 5% CO2 incubator. Passaging was performed by brief (3-5 minute) treatment with 0.5 mM EDTA and 0.25X TRYPLE Express (Gibco) in phosphate-buffered saline (PBS), followed by pipetting to break the colonies into small clumps. Cells were treated with 10 µM Y-27632 for 24 hours after each passage. Cells were tested every three months for mycoplasma using the ATCC Universal Mycoplasma Detection Kit. All cells tested negative. For experiments requiring single cell dissociation and counting, cells were harvested with Accutase and counted using trypan blue staining with a Countess II automated cell counter (Thermo Fisher).

Generation and verification of reporter lines

Knock-in donor plasmids targeting *REC8* and *SYCP3* were constructed by Gibson assembly of 5' and 3' homology arms, an insert containing a fluorescent marker joined to the gene of interest by a T2A linker, and a plasmid backbone containing an MC1-DTA marker to select against random integration. sgRNA oligos were cloned into pX330 (Addgene #42230), which also expresses Cas9. Oligo sequences are provided in Supplementary Table 4. All plasmids will be made available on Addgene following acceptance of this paper.

Knock-in electroporations were performed as previously described (*25*). In summary, 1 µg donor plasmid and 1 µg sgRNA/Cas9 plasmid were co-electroporated into 200,000 hiPSCs using a Lonza Nucleofector with 20 µL P3 solution and pulse setting CA-137. Colonies were picked after selection and genotyped by PCR. Successful knockin, absence of off-target edits, and euploidy of the reporter lines were all confirmed by whole genome sequencing (Novogene, 10X coverage) and SeqVerify computational analysis (*18*). Low-passage cells were cryopreserved using CryoStor CS10 (Stemcell Technologies) and banked for future use.

Functional validation of the reporter alleles was performed by CRISPRa. For each allele, three CRISPRa plasmids were constructed, each containing a sgRNA targeting the promoter as well as a doxycycline-inducible expression cassette for dCas9-VPR (*28*). Equimolar mixtures of plasmids (1 µg total) were electroporated into 200,000 hiPSCs using a Lonza Nucleofector with 20 µL P3 solution and pulse setting CA-137. After two days, cells were harvested with Accutase and analyzed by flow cytometry (Fig. S1).

bioRxiv preprint doi: https://doi.org/10.1101/2024.05.31.596483; this version posted June 1, 2024. The copyright holder for this preprint (which was not certified by peer review) is the author/funder, who has granted bioRxiv a license to display the preprint in perpetuity. It is made available under aCC-BY 4.0 International license.

Identification of candidate meiosis-promoting factors

We obtained a human fetal germ cell scRNAseq dataset from Garcia-Alonso *et al.* 2022 (https://www.reproductivecellatlas.org/gonads.html) (*16*). We performed pySCENIC analysis (*29*), which involves inferring a gene regulatory network, finding transcription factor (TF) regulons based on known binding motifs, and ranking regulon activity in each cell type. We chose 21 TFs to screen based on high activity in STRA8+ and/or meiotic oogonia. Because regulon analysis ignores non-TF factors, including RNA-binding proteins, we selected 18 additional factors to screen based on a gene regulatory network analysis. In this analysis, we found differentially expressed genes between meiotic oogonia and all other cell types, then calculated which genes in the regulatory network were upstream of the differentially expressed genes, multiplying network edge weights by differential expression fold-changes to calculate a weighted score. We included a further 12 factors based on literature reports of pro-meiotic function. Finally, we included 27 factors from the Cancer Pathways ORFs library (*30*), which contains modulators of several common cellular signaling pathways. Our initial library for barcode enrichment screening contained 78 factors. For scRNAseq screening, we included an additional 10 factors involved in epigenetics and signal transduction, bringing the total to 88. A full list of the factors in our library is provided in Supplementary Table 2.

Plasmid library construction and PiggyBac transposon integration

Expression plasmids for 88 candidate regulatory factors were constructed by MegaGate cloning (*31*) into a barcoded PiggyBac destination vector containing a doxycycline-inducible promoter (*28*), as well as a puromycin selection marker. Barcodes and transgene sequences were verified by Sanger sequencing. For later experiments, an alternative version of the plasmid lacking the barcode and containing a hygromycin selection marker was constructed for top candidate factors. For constitutive BCL2 expression, a version of the plasmid was constructed with an EF1a promoter instead of a doxycycline-inducible promoter. All plasmids will be made available on Addgene following acceptance of this paper.

Plasmids were pooled and co-electroporated into iPSCs along with a PiggyBac transposase expression plasmid (Systems Bioscience), using a Lonza Nucleofector with pulse setting CA-137. For medium-copy (3–5 per cell) integration, 5 fmol of pooled library and 500 ng of transposase plasmid were used per 200,000 hiPSCs and 20 µL of P3. For high-copy (10–50 per cell) integration, 50 fmol of pooled library was used instead. Average integration numbers were previously characterized for the same transposons used in this study (*28*), but not evaluated directly. Selection was performed with puromycin (400 ng/mL) and/or

bioRxiv preprint doi: https://doi.org/10.1101/2024.05.31.596483; this version posted June 1, 2024. The copyright holder for this preprint (which was not certified by peer review) is the author/funder, who has granted bioRxiv a license to display the preprint in perpetuity. It is made available under aCC-BY 4.0 International license.

hygromycin (50 μg/mL) beginning 2 days after nucleofection and continuing for at least 5 additional days before subsequent experiments.

Flow cytometry

Flow cytometry was performed on a BD LSR Fortessa instrument. Cell sorting was performed on a Sony SH800S sorter using a 100 μm chip. Compensation controls were acquired using cells in which mGreenLantern and tdTomato reporters had been activated by CRISPRa. DAPI (100 ng/mL) was used to stain dead cells for exclusion. Data analysis was performed using the Cytoflow python package (v. 1.0.0) (*32*). Representative gating is shown in Fig. S3A.

Immunofluorescence microscopy

Cells were cultured on Matrigel-coated ibidi dishes (8-well, cat# 80826; or 96-well, cat# 89626). For 8-well dishes, 100 μL of staining solutions and 200 μL of wash solutions were used per well. For 96-well dishes, 50 μL of staining solutions and 100 μL of wash solutions were used per well. All steps except primary antibody incubation were performed at room temperature.

Cells were washed with PBS and fixed with 4% PFA in PBS for 10 minutes, followed by one 5-minute wash with PBS and one 5-minute wash with PBST (0.1% Triton X-100 in PBS). Cells were incubated with blocking solution (1% BSA and 5% normal donkey serum in PBST) for 20-30 minutes, followed by an overnight incubation at 4 °C with primary antibodies in blocking solution. Three 5-minute PBST washes were performed, followed by a 1-hour incubation with secondary antibodies and DAPI (1 μg/mL) in blocking solution. Two 5-minute PBST washes were performed, and the cells were stored in the dark at 4°C in PBST prior to imaging on a Zeiss LSM980 confocal microscope using a LD C-Apochromat 40x/1.1 water immersion objective. A list of antibodies used, and their dilutions, is provided in Supplementary Table 5.

Initial image processing was performed using FIJI (ImageJ version 2.14.0/1.54f) (*33*). Brightness and contrast were adjusted equally across all images from each experiment. Segmentation and quantification were performed using the cellpose Python package (version 2.2.3) (*34*). Thresholding was performed by using a negative control (typically hiPSCs) to determine the average background staining intensity, subtracting this average background intensity from all regions, then classifying any region that was >2x brighter than then 95th percentile of the negative control as positive. Analysis code is available on Github: https://github.com/mpiersonsmela/meiosis

Barcode enrichment screening

bioRxiv preprint doi: https://doi.org/10.1101/2024.05.31.596483; this version posted June 1, 2024. The copyright holder for this preprint (which was not certified by peer review) is the author/funder, who has granted bioRxiv a license to display the preprint in perpetuity. It is made available under aCC-BY 4.0 International license.

Reporter iPSCs containing integrated expression transposon vectors were harvested with Accutase. Cells were seeded on Matrigel-coated 6-well plates (500,000 cells per plate) in mTeSR1 medium (Stemcell Technologies) with doxycycline (1 µg/mL) and Y-27632 (10 µM). A media change was performed after 24 hours. At this point, candidate differentiation media were tested, based on previous claims of meiosis induction in the literature. These included:

A-MEM with 1X Glutamax, 1X Insulin-Transferrin-Selenium-X supplement, 0.2% BSA, 0.2% chemically defined lipid concentrate, 200 µg/mL ascorbic acid, 1 ng/mL FGF2, and 20 ng/mL GDNF (*35*).

Nutrient restriction/retinoic acid: EBSS with 0.1X IMDM, 0.1X supplements (N2, glutamax, sodium pyruvate, MEM essential vitamins, non-essential amino acids), 0.1% FBS, 0.5% Knockout Serum Replacement, 0.6 mg/mL glucose, 0.1 mg/mL lactic acid, 0.5 mg/mL BSA, 5 µM 2-mercaptoethanol, 10 µM ascorbic acid, 1 µg/mL biotin, 3 ng/mL beta-estradiol, 1 ng/mL FGF2, 1.5 ng/mL GDNF, and 100 nM retinoic acid (*7*).

We additionally tested mTeSR1 (which maintains primed pluripotency), HENSM (which induces naïve pluripotency) (*36*), StemPro-34 based spermatogonial stem cell culture medium (*37*), and APEL2 (Stemcell Technologies; a "neutral" medium lacking growth factors).

The cells were cultured in these six media, all supplemented with 1 µg/mL doxycycline, for six additional days. A media change was performed each day. Cells were harvested with Accutase on day seven post-induction, and reporter-positive cells were isolated by FACS. DNA was extracted from REC8+, SYCP3+, and DDX4+ cells, as well as the pre-sorting population and pre-differentiation population. Barcodes were amplified by two rounds of PCR and sequenced on an Illumina MiSeq as previously described (*25*). Barcode enrichment was calculated by comparing the barcode frequencies in reporter-positive and pre-sorting cells. We also sequenced barcodes from the pre-differentiation cells, but did not use these for analysis since effects were dominated by changes in cell growth rate.

CRISPRi of epigenetic factors

Guide RNAs targeting the promoters of ten epigenetic factors (*MAX, MGA, E2F6, RNF2, PCGF6, SETDB1, DNMT1, DNMT3A, DNMT3B,* and *UHRF1*; three guides per gene) were cloned into a doxycycline-inducible dCas9-KRAB expression transposon plasmid (*28*). Each transposon plasmid was integrated into SYCP3 reporter iPSCs as described above. iPSCs were treated with 1 µg/mL doxycycline in mTeSR1 for six days, harvested with Accutase, and analyzed by flow cytometry (Fig. S4). Additionally, knockdown efficiency was

bioRxiv preprint doi: https://doi.org/10.1101/2024.05.31.596483; this version posted June 1, 2024. The copyright holder for this preprint (which was not certified by peer review) is the author/funder, who has granted bioRxiv a license to display the preprint in perpetuity. It is made available under aCC-BY 4.0 International license.

evaluated by qPCR with PowerUp SYBR Green Master Mix (Thermo Fisher) using *GAPDH* as a control gene (Fig. S4C). Primers used are given in Supplementary Table 4.

Screening of conditions for reporter activation

Several flow cytometry experiments were performed in order to optimize conditions for activating REC8 and SYCP3 expression in male (PGP1) and female (F3) reporter lines.

First, expression vectors for sixteen promising factors (Fig. S3) chosen based on barcode enrichment data were individually integrated into REC8 and SYCP3 reporter iPSC lines, and expression was induced by treatment with 1 μg/mL doxycycline in APEL2 medium following the protocol described above in the barcode enrichment section.

Second, a fractional factorial screen for REC8 activation was conducted in F3 and PGP1 D4TR8G reporter lines, using 32 combinations of seven promising factors and following the same differentiation protocol. (Fig. 1C)

Third, REC8, SYCP3, and DDX4 activation was measured after performing differentiation using expression of STRA8, HOXB5, and BCL2 in the presence of different basal media and additives. Four basal media were tested: mTeSR1 (Stemcell Technologies); APEL2 (Stemcell Technologies); DMEM/F12 with 1X Glutamax and 10% KSR (Gibco); and Advanced RPMI with 1X nonessential amino acids, 1X Glutamax, and 0.5X B27 supplement minus Vitamin A (all Gibco). For each of the differentiation media, five additives were tested, as well as a doxycycline-only control. The additives and their concentrations were: 1 μM retinoic acid, 1 μM AM580, 25 μM PRT4165, 20 μM RB3, and 1 mM sodium valproate. A media change was performed every day. At days 6, 7, and 8 post-induction, cells were harvested with Accutase and analyzed by flow cytometry (Fig. 1D)

Fourth, REC8, SYCP3, and DDX4 activation was measured in cells treated with or without DNMT1 inhibitor (5 μM GSK3484862). Differentiation was performed using expression of STRA8, HOXB5, and BCL2 in APEL2 medium supplemented with 1 μM AM580 and 1 μg/mL doxycycline. Cells were harvested with Accutase after 7 days and analyzed by flow cytometry (Fig. 1E).

scRNAseq screening

Prior to scRNAseq, the following six cell populations were generated by integration of transposon expression vectors:

- PGP1 D4TR8G, with BCL2, HOXB5, and STRA8 under hygromycin selection
- F3 D4TS3G, with BCL2, HOXB5, and STRA8 under hygromycin selection

bioRxiv preprint doi: https://doi.org/10.1101/2024.05.31.596483; this version posted June 1, 2024. The copyright holder for this preprint (which was not certified by peer review) is the author/funder, who has granted bioRxiv a license to display the preprint in perpetuity. It is made available under aCC-BY 4.0 International license.

- PGP1 D4TR8G, with BCL2, HOXB5, and STRA8 under hygromycin selection and the full pool of 88 candidate factors under puromycin selection
- F3 D4TS3G, with BCL2, HOXB5, and STRA8 under hygromycin selection and the full pool of 88 candidate factors under puromycin selection
- PGP1 D4TR8G, with the full pool of 88 candidate factors under puromycin selection
- F3 D4TS3G, with the full pool of 88 candidate factors under puromycin selection

The cells were differentiated according to the following method, which had been chosen based on its ability to activate REC8 and SYCP3 expression. Cells containing integrated expression vectors were seeded in mTeSR1 containing 10 μM Y-27632, 5 μM GSK3484862, and 1 μg/mL doxycycline. After 24 hours, the medium was changed to APEL2 with 5 μM GSK3484862, 1 μM AM580, and 1 μg/mL doxycycline. A media change was performed every other day. After a total of seven days of differentiation, cells were harvested with Accutase and sorted based on reporter expression. Cells were fixed using a Parse fixation kit, and scRNAseq library preparation was performed using a Parse WT Mega v2 kit. A list and description of samples is provided in Supplementary Table 6. Sequencing was performed on an Illumina Novaseq X Plus using three full 10B PE150 flowcells. Alignment and counts matrix generation was performed using the Parse Biosciences pipeline (v.0.9.6).

To enrich the library for barcode sequences, we first performed PCR with biotinylated primers to generate a dsDNA biotinylated bait containing the 120bp of sequence immediately 3' of the barcode sequence in our expression vector. We isolated the bait DNA from the PCR using a 3X volume of ProNex beads, and eluted in 10 mM Tris pH 8.0 buffer. Next, we used 200 fmol of bait DNA as a custom probe in the Parse Gene Capture kit, following the manufacturer's protocol aside from this substitution. After qPCR to verify the barcode enrichment, the resulting library was sequenced on one lane of a NovaSeq X Plus 10B PE150 flowcell.

scRNAseq data were filtered by number of reads per cell (<100,000), number of genes detected (>1,000 and <14,000), and mitochondrial read percentage (<10%). Transgene barcode reads were merged into the dataset by matching cell barcodes. Analysis was performed using scanpy (*38*) for normalization, integration with the fetal germ cell reference atlas, and gene scoring (*16*).

Refinement of factors for meiosis induction

Several experiments were performed in order to optimize the protocol for meiosis induction. First, for each of the 23 candidate factors identified by the scRNAseq screen (excluding HOXB5, which was already integrated), an expression vector with a puromycin selection marker was integrated into PGP1 D4TR8G and F3 D4TS3G reporter hiPSCs which

bioRxiv preprint doi: https://doi.org/10.1101/2024.05.31.596483; this version posted June 1, 2024. The copyright holder for this preprint (which was not certified by peer review) is the author/funder, who has granted bioRxiv a license to display the preprint in perpetuity. It is made available under aCC-BY 4.0 International license.

already contained expression vectors for BCL2, HOXB5, and STRA8 with hygromycin selection markers. Two control conditions were additionally included: BCL2, HOXB5, and STRA8 only; and a no-factor control. Cells were differentiated using the same conditions as for the scRNAseq experiment (APEL2 with doxycycline, AM580, and GSK3484862). Cells were fixed and stained for SYCP3, HORMAD1, and DDX4 after 7 days of differentiation.

Second, expression vectors for the seven top factors identified in the previous experiment (BCL2, HOXB5, STRA8, myr-AKT1, BOLL, MEIOC, and MEIOSIN) were pooled and integrated into PGP1 D4TR8G and F2 D4TDZG hiPSCs. Cells were differentiated in the same manner, and fixation and staining for SYCP3, HORMAD1, and γH2AX was performed after 7, 9, 13, and 16 days of differentiation. As a control, the same differentiation and staining was performed using only BCL2, HOXB5, and STRA8.

Third, a fractional factorial screen was performed in order to identify the contributions of the seven top factors. Sixteen combinations of the seven factors (including one control combination lacking all factors) were integrated into F2 D4TDZG, F3 D4TS3G, and PGP1 D4TR8G reporter lines. Additionally, expression vectors for DAZL and BOLL were integrated in order to evaluate previous claims that those factors alone could induce meiosis (*11*, *14*). Cells were fixed and stained for SYCP3, HORMAD1, and γH2AX after 13 days of differentiation. Additional cells were analyzed by flow cytometry for reporter expression.

Fourth, a full factorial screen was performed in order to confirm the best factors for meiosis induction. A constitutive EF1a-driven BCL2 expression plasmid was integrated into F2 D4TDZG, F3 D4TT2G, and PGP1 D4TR8G reporter hiPSCs under hygromycin selection. Then, all eight possible combinations of HOXB5, BOLL, and MEIOC expression vectors were integrated under puromycin selection. Cells were fixed and stained for after 13 days of differentiation, with the first 3 days at 37 °C and the remainder at 34 °C. Two stains were used: SYCP3, HORMAD1, and γH2AX; and TEX12 and SYCP3.

Timing of media additives and factor expression:

A Shield1-inducible HOXB5 PiggyBac transposon plasmid was constructed using an EF1a promoter driving HOXB5 with a C-terminal degradation domain, which could be stabilized by addition of Shield1. This plasmid, along with expression plasmids for constitutive EF1a-driven BCL2 and doxycycline-inducible BOLL, were integrated into F2 D4TDZG, F3 D4TS3G, and PGP1 D4TR8G reporter lines. Cells were seeded at a density of 50,000/cm^2 in Matrigel coated 96-well ibidi plates in mTESR1 + 10 µM Y-27632. After 24 hours, the medium was changed to APEL2. Subsequently, a full media change was performed every 48 hours, and cells were fixed and stained after 11 days of differentiation. This experiment was performed at 37 °C.

bioRxiv preprint doi: https://doi.org/10.1101/2024.05.31.596483; this version posted June 1, 2024. The copyright holder for this preprint (which was not certified by peer review) is the author/funder, who has granted bioRxiv a license to display the preprint in perpetuity. It is made available under aCC-BY 4.0 International license.

The following media additive conditions were tested:

1. No additives (negative control)
2. Full, continuous dose of all additives (positive control): 5 μM GSK3484862 (DNMT1i), 1 μM AM580, 1 μg/mL doxycycline, 500 nM Shield1
3. No doxycycline, 100 nM Shield1, full dose of DNMTi and AM580
4. 0.1 μg/mL doxycycline, no Shield1, full dose of DNMTi and AM580
5. 0.1 μg/mL doxycycline, 100 nM Shield1, full dose of DNMTi and AM580
6. 0.1 μg/mL doxycycline, 500 nM Shield1, full dose of DNMTi, and AM580
7. 1 μg/mL doxycycline, 100 nM Shield1, full dose of DNMTi and AM580
8. Full dose of all additives except doxycycline
9. Doxycycline added only after day 1. Other additives at full dose.
10. Doxycycline added only after day 3. Other additives at full dose.
11. Doxycycline added only after day 5. Other additives at full dose.
12. Doxycycline added only after day 7. Other additives at full dose.
13. Doxycycline added only after day 9. Other additives at full dose.
14. Full dose of all additives except Shield1
15. Shield1 added only after day 1. Other additives at full dose.
16. Shield1 added only after day 3. Other additives at full dose.
17. Shield1 added only after day 5. Other additives at full dose.
18. Shield1 added only after day 7. Other additives at full dose.
19. Shield1 added only after day 9. Other additives at full dose.
20. Full dose of all additives except AM580
21. AM580 added only after day 1. Other additives at full dose.
22. AM580 added only after day 3. Other additives at full dose.
23. AM580 added only after day 5. Other additives at full dose.
24. AM580 added only after day 7. Other additives at full dose.
25. AM580 added only after day 9. Other additives at full dose.
26. Full dose of all additives except DNMTi
27. DNMTi withdrawn after day 5. Other additives at full dose.
28. DNMTi withdrawn after day 7. Other additives at full dose.
29. DNMTi withdrawn after day 9. Other additives at full dose.
30. Shield1 added only from days 0–5, doxycycline added only from days 5–11, other additives at full dose.
31. Shield1 added only from days 0–7, doxycycline added only from days 5–11, other additives at full dose.
32. Shield1 added only from days 0–7, doxycycline added only from days 7–11, other additives at full dose.

bioRxiv preprint doi: https://doi.org/10.1101/2024.05.31.596483; this version posted June 1, 2024. The copyright holder for this preprint (which was not certified by peer review) is the author/funder, who has granted bioRxiv a license to display the preprint in perpetuity. It is made available under aCC-BY 4.0 International license.

Evaluation of temperatures and timings for meiosis induction

As an initial experiment to evaluate the effects of lower temperature on male meiosis, PGP1 D4TR8G reporter hiPSCs containing integrated expression vectors for BCL2, HOXB5, BOLL, and MEIOC were seeded in 8-well ibidi dishes at 50,000 cells/cm2 in mTeSR1 with 5 μM GSK3484862, 1 μg/mL doxycycline, and 10 μM Y-27632. After 24 hours, the media was replaced with APEL2 containing 5 μM GSK3484862 and 1 μg/mL doxycycline. A 50% media change was performed every 2 days, and GSK3484862 was withdrawn starting on day 7. Initially, all cells were cultured at 37 °C. One plate was moved to a 34 °C incubator after the first day. Cells were fixed on day 13, stained for HORMAD1, SYCP3, and γH2AX, and imaged.

As a confirmatory experiment, F2 D4TDZG, F3 D4TS3G, and PGP1 D4TR8G reporter hiPSCs containing integrated expression vectors for BCL2, HOXB5, BOLL, and MEIOC were differentiated according to the same protocol. The following conditions were tested:

1. 34 °C starting on day 3, fixation at day 13
2. 34 °C starting on day 1, fixation at day 14
3. 34 °C starting on day 3, fixation at day 15
4. 34 °C starting on day 3, fixation at day 16
5. Continuous 37 °C, fixation at day 16
6. 34 °C starting on day 1, fixation at day 17
7. 34 °C starting on day 3, fixation at day 19
8. 34 °C starting on day 3, fixation at day 21

After fixation, cells were stained for HORMAD1, SYCP3, γH2AX, and actin, and imaged.

Final protocol for meiosis induction

Based on the results of our screening and optimization experiments, which are described above, we have developed the following protocol for robust initiation of meiosis:

A constitutive or doxycycline-inducible expression vector for the anti-apoptotic factor BCL2, as well as doxycycline-inducible expression vectors for meiosis-promoting factors (HOXB5, BOLL, and/or MEIOC), are integrated into human iPSCs using PiggyBac transposase. The iPSCs are seeded at 50,000 cells/cm^2 on Matrigel-coated plates in mTeSR1 supplemented with 5 μM GSK3484862, 1 μg/mL doxycycline, and 10 μM Y-27632. For 6-well plates, 1.5 mL of medium is used per well, and volumes for smaller plates are scaled down proportionally to their surface area. After one day, the media is replaced with APEL2 containing 5 μM GSK3484862 and 1 μg/mL doxycycline. A 50% media change is performed every 2 days, and GSK3484862 is withdrawn starting on day 7. Initiation of meiosis is complete by roughly day 13.

bioRxiv preprint doi: https://doi.org/10.1101/2024.05.31.596483; this version posted June 1, 2024. The copyright holder for this preprint (which was not certified by peer review) is the author/funder, who has granted bioRxiv a license to display the preprint in perpetuity. It is made available under aCC-BY 4.0 International license.

Timecourse scRNAseq and imaging

Plasmids for constitutive expression of BCL2 and doxycycline-inducible expression of HOXB5, BOLL, and MEIOC were integrated into F2 D4TDZG, F3 D4TT2G, and PGP1 D4TR8G reporter hiPSCs. Meiosis was initiated following the final optimized protocol, with cells cultured on 8-well ibidi dishes for immunofluorescence imaging and 12-well plates for scRNAseq. At each day from day 0 (hiPSC) to day 15, cells were fixed for imaging and harvested for scRNAseq. Stains used for imaging were: rabbit anti-HORMAD1, goat anti-SYCP3, mouse anti γH2AX, and rat anti-T2A; and rabbit anti-TEX12, goat anti-SYCP3, mouse anti-RAD51, and rat anti-KI67. Samples for scRNAseq were counted and fixed using the Parse Biosciences fixation kit. Library preparation was performed using the Parse Biosciences WT v3 kit. Sequencing was performed on two lanes of a Novaseq X Plus 25B PE150 flowcell. Alignment and counts matrix generation was performed using the Parse Biosciences pipeline (v.1.2.1). Filtering, normalization, and cell type annotation were performed in scanpy as described above. To build a reference atlas, we combined the fetal gonad atlas and adult testis atlas (*16*, *22*), removing somatic cell types from the testis atlas and only keeping genes that were expressed in both atlases.

Statistics and data analysis

Results of fractional factorial screens were analyzed by fitting linear models using the lm function in R (version 4.3.2). For flow cytometry data, values expressed as a proportion (0 - 100%) were logit-transformed before fitting the model. Significance calculations in bar plots were performed using two-tailed Mann-Whitney U tests.

bioRxiv preprint doi: https://doi.org/10.1101/2024.05.31.596483; this version posted June 1, 2024. The copyright holder for this preprint (which was not certified by peer review) is the author/funder, who has granted bioRxiv a license to display the preprint in perpetuity. It is made available under aCC-BY 4.0 International license.

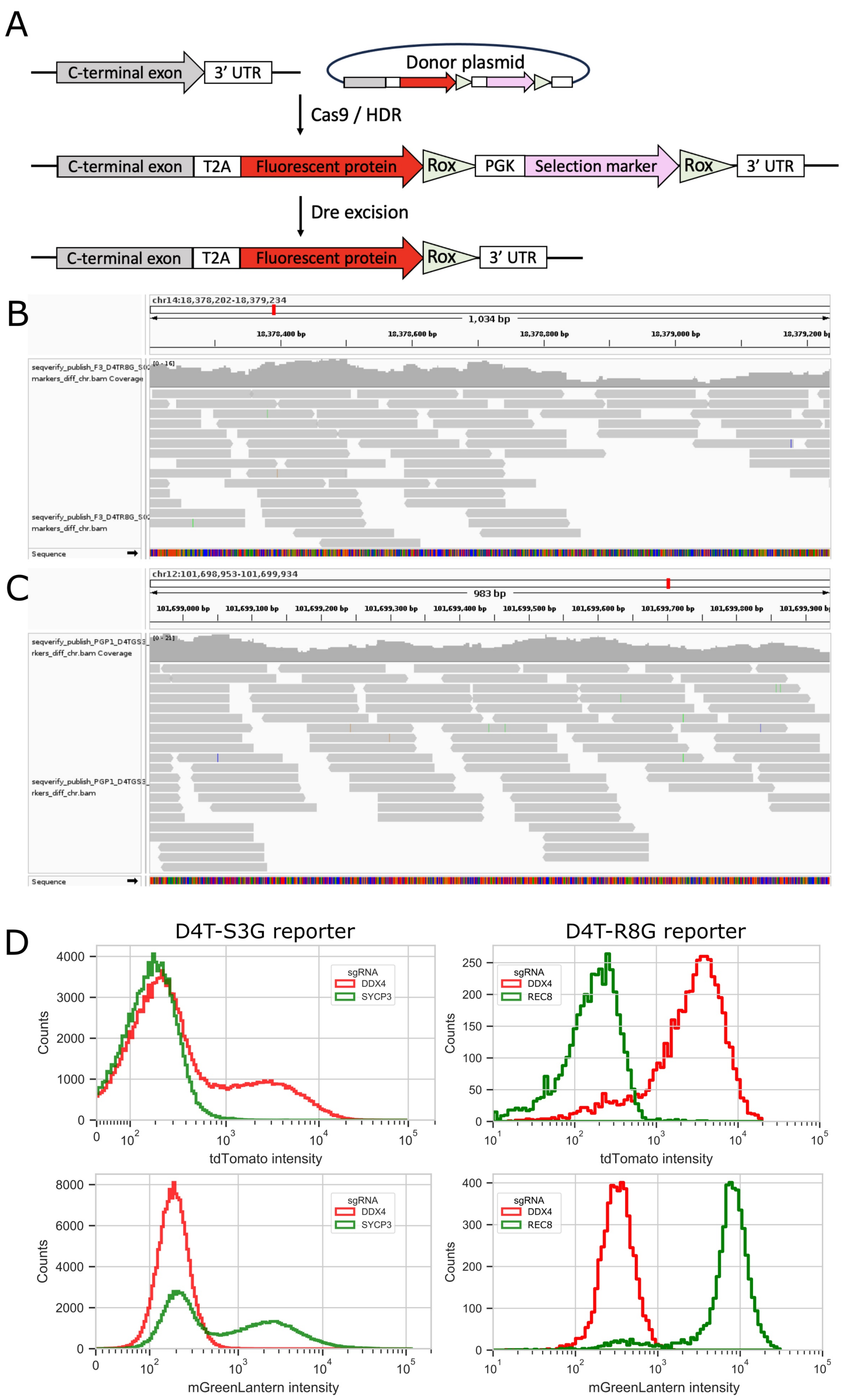

Fig. S1. Construction and validation of hiPSC reporter lines. (**A**) Knock-in editing strategy. (**B**) SeqVerify validation of REC8 reporter allele using whole genome sequencing. (**C**) SeqVerify validation of SYCP3 reporter allele using whole genome sequencing. (**D**) Functional validation of reporter hiPSCs using flow cytometry and CRISPRa with gRNAs targeting the promoters of *REC8, SYCP3*, and *DDX4*.

bioRxiv preprint doi: https://doi.org/10.1101/2024.05.31.596483; this version posted June 1, 2024. The copyright holder for this preprint (which was not certified by peer review) is the author/funder, who has granted bioRxiv a license to display the preprint in perpetuity. It is made available under aCC-BY 4.0 International license.

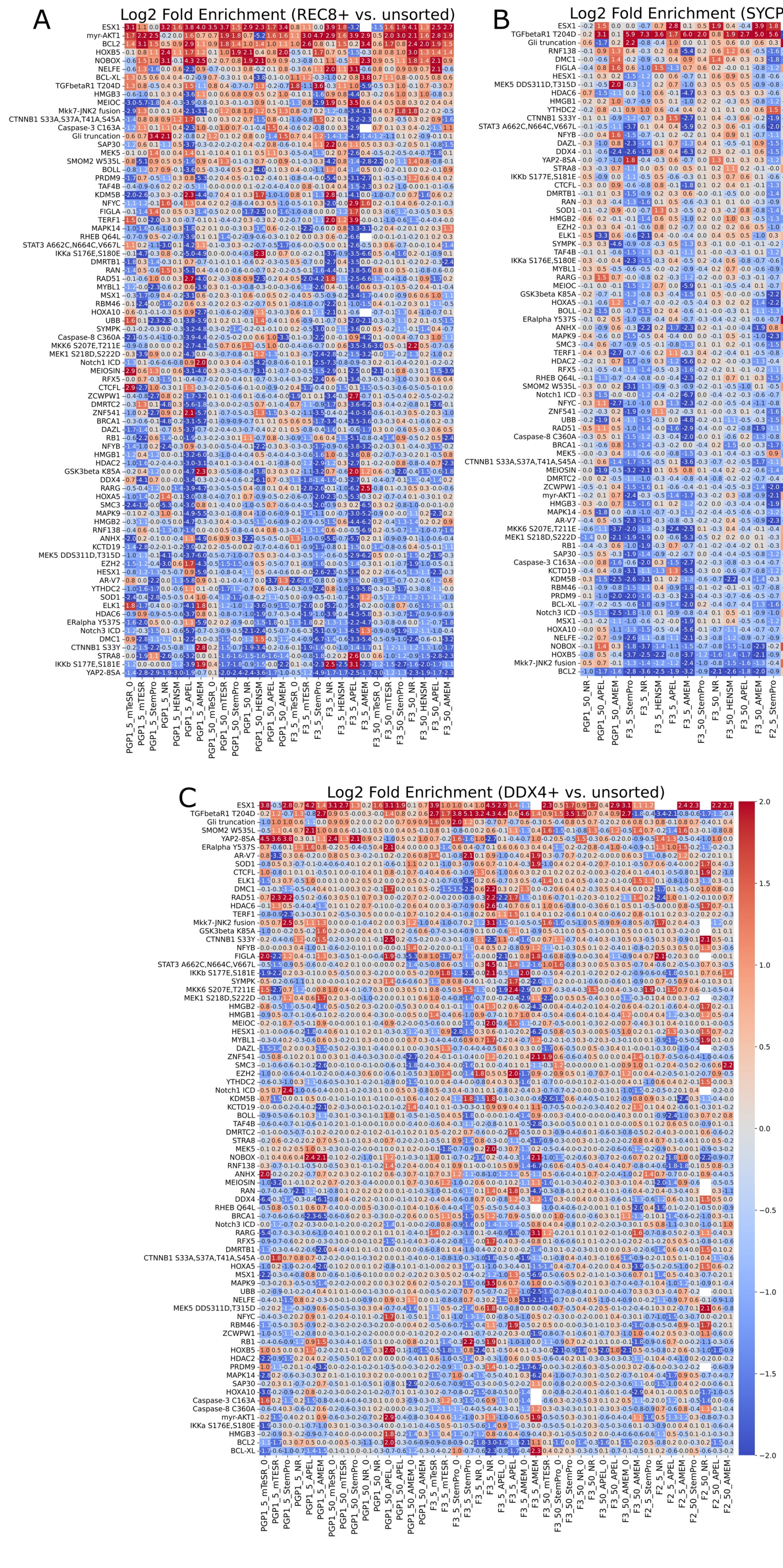

(caption on following page)

bioRxiv preprint doi: https://doi.org/10.1101/2024.05.31.596483; this version posted June 1, 2024. The copyright holder for this preprint (which was not certified by peer review) is the author/funder, who has granted bioRxiv a license to display the preprint in perpetuity. It is made available under aCC-BY 4.0 International license.

Fig. S2. Barcode enrichment results for (**A**) REC8, (**B**) SYCP3, and (**C**) DDX4. Reporter hiPSCs (F2, F3, and PGP1) were nucleofected with low (5 fmol) or high (50 fmol) doses of plasmid library pool, treated with doxycycline to induce expression, and differentiated in various media (mTeSR, StemPro, nutrient restriction (NR), HENSM, spermatogonial stem cell medium (AMEM), and APEL2). Reporter-positive cells were sorted after 7 days, and barcode frequencies were compared to unsorted cells.

bioRxiv preprint doi: https://doi.org/10.1101/2024.05.31.596483; this version posted June 1, 2024. The copyright holder for this preprint (which was not certified by peer review) is the author/funder, who has granted bioRxiv a license to display the preprint in perpetuity. It is made available under aCC-BY 4.0 International license.

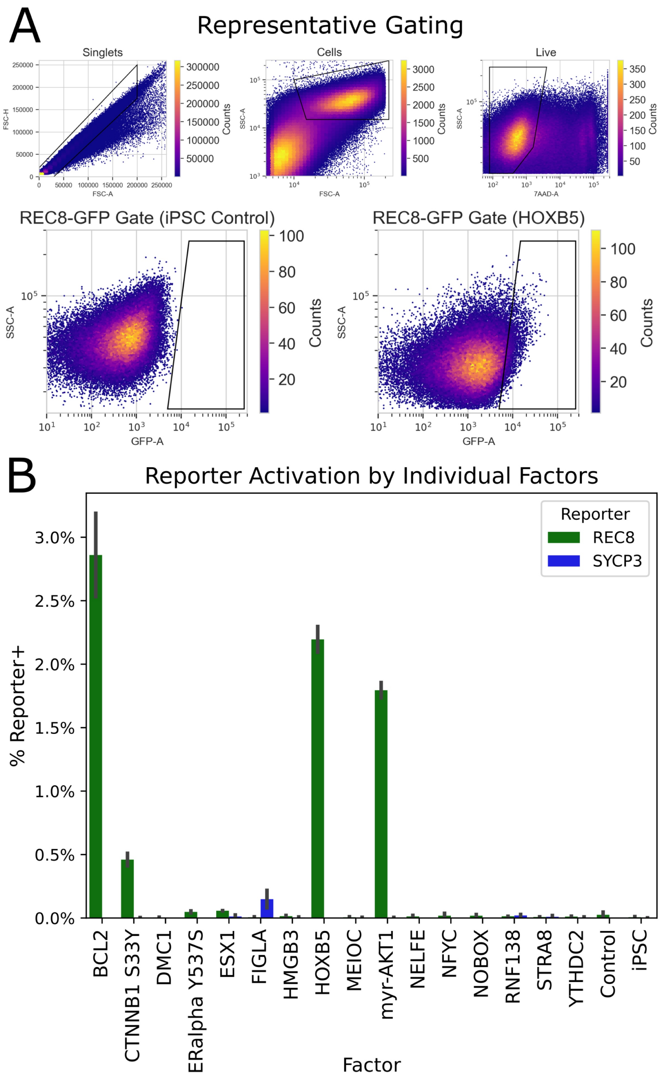

Fig. S3. Flow cytometry analysis of reporter expression induced by individual factors. (**A**) Representative gating strategy for singlets, cells, live cells, and reporter-positive cells. (**B**) Activation of REC8 and SYCP3 reporters by sixteen individual factors chosen based on barcode enrichment results (n = 2 biological replicates per factor per reporterX). Error bars are standard error of the mean.

bioRxiv preprint doi: https://doi.org/10.1101/2024.05.31.596483; this version posted June 1, 2024. The copyright holder for this preprint (which was not certified by peer review) is the author/funder, who has granted bioRxiv a license to display the preprint in perpetuity. It is made available under aCC-BY 4.0 International license.

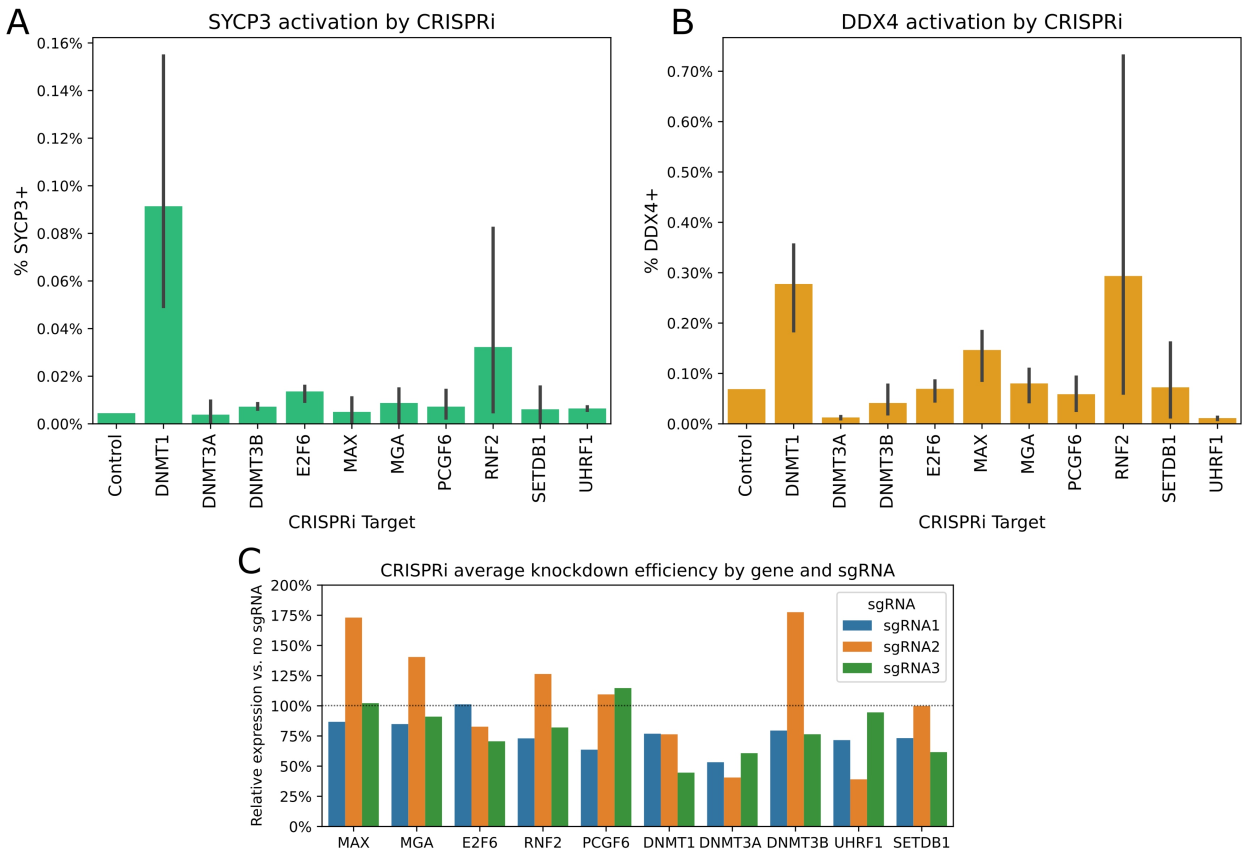

Fig. S4. Pilot screen for activation of SYCP3 and DDX4 expression upon CRISPRi knockdown of ten epigenetic modifiers. (**A**) Activation of SYCP3 expression measured by flow cytometry (n = 3 sgRNAs per gene). (**B**) Activation of DDX4 expression measured by flow cytometry (n = 3 sgRNAs per gene). (**C**) qPCR measurement of average knockdown efficiency (n = 2 technical replicates per guide), calculated by $2^{-\Delta\Delta Ct}$ with *GAPDH* as a reference gene. The bulk knockdown efficiency was poor for most guides, although it is possible that subpopulations of cells experienced a greater knockdown.

bioRxiv preprint doi: https://doi.org/10.1101/2024.05.31.596483; this version posted June 1, 2024. The copyright holder for this preprint (which was not certified by peer review) is the author/funder, who has granted bioRxiv a license to display the preprint in perpetuity. It is made available under aCC-BY 4.0 International license.

A

	Factor	DDX4 Z-score	REC8 Z-score	SYCP3 Z-score	HORMAD1 Z-score	Weighted Ranking
1	BOLL	1.52	0.32	1.02	1.66	10.00
2	myr-AKT1	-0.76	0.22	-1.14	2.72	9.75
3	MEIOSIN	-1.14	0.37	0.54	1.28	6.46
4	MEIOC	0.08	0.37	-0.26	1.04	5.37
5	ESX1	1.57	-0.90	2.97	0.12	5.30
6	DAZL	1.21	0.32	0.79	0.27	4.43
7	TET3	-0.35	-0.11	0.97	0.46	3.88
8	DDX4	-0.41	0.36	-0.63	0.54	2.78
9	RBM46	0.38	0.36	0.06	0.11	2.51
10	TET1	0.10	0.34	-0.24	0.11	1.97
11	PAX5	0.56	-0.97	1.65	-0.17	1.93
12	HDAC2	0.33	0.32	-0.40	0.11	1.88
13	KDM5B	0.76	0.32	-0.02	-0.17	1.57
14	YTHDC2	-0.16	0.34	-0.37	0.06	1.45
15	DMRTC2	0.55	0.34	0.23	-0.47	0.66
16	HOXB5	0.00	0.33	-0.25	-0.44	-0.15
17	NOBOX	-0.11	0.37	-0.61	-0.44	-0.61
18	DMC1	0.68	0.33	0.23	-1.01	-1.21
19	PRDM9	-0.95	0.37	-1.44	-0.30	-1.60
20	BRCA1	0.22	0.34	-0.59	-0.77	-1.63
21	RAD51	0.06	0.08	0.18	-1.01	-1.96
22	MYBL1	-0.21	0.34	-0.93	-1.04	-3.27
23	SOX17	-2.14	0.22	-1.60	-1.54	-7.24
24	MKK6	-1.80	-4.37	-0.15	-1.30	-10.00
Ctrl	Ctrl	-0.24	0.29	1.12	0.25	n/a

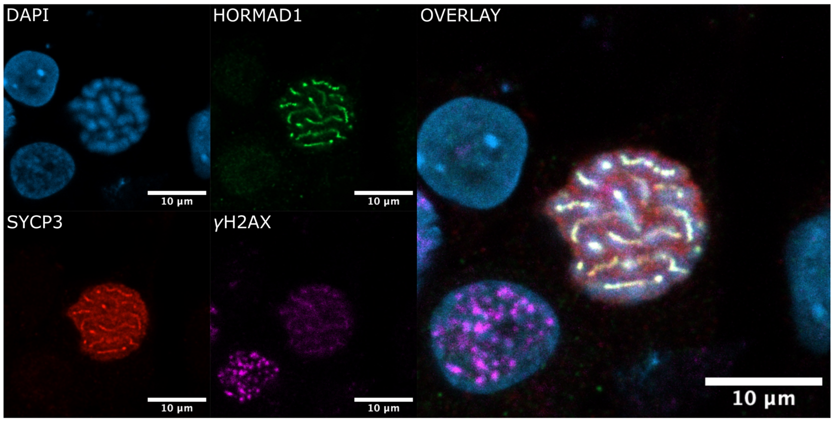

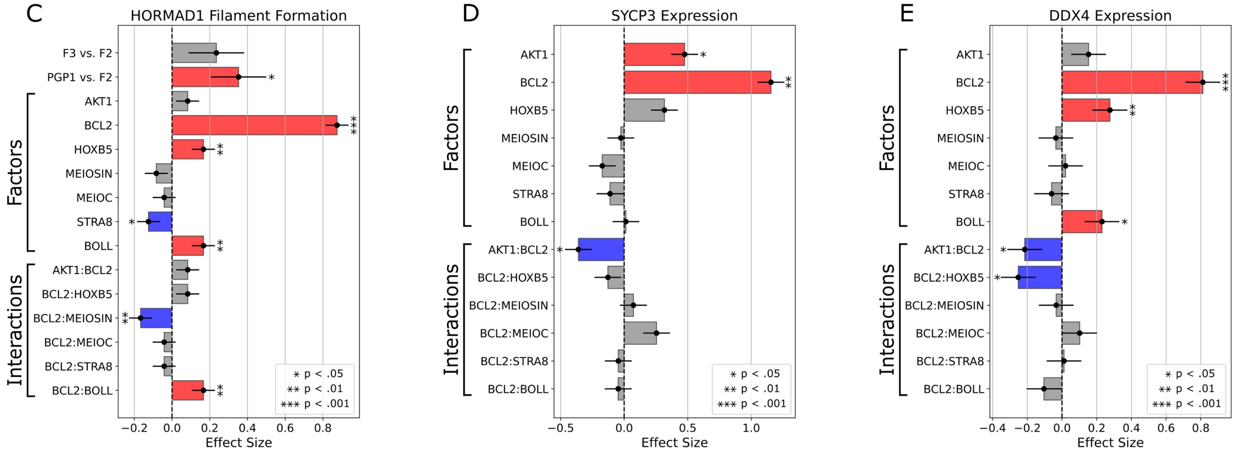

Fig. S5. Optimization of factors for meiosis induction. (**A**) 24 candidate factors identified by an scRNAseq screen (Fig. 2D) were each co-expressed with STRA8, BCL2, and HOXB5. Reporter expression was analyzed by flow cytometry, and HORMAD1 expression was analyzed by immunofluorescence microscopy. Factors were ranked according to the results. (**B**) HORMAD1 and SYCP3 filament formation observed by immunofluorescence microscopy after twelve days of expression of seven top factors (STRA8, BCL2, HOXB5, BOLL, AKT1, MEIOSIN, and MEIOC). (**C**) Results of a fractional factorial screen of the seven top factors, for HORMAD1 filament formation. (**D**) Results for SYCP3 expression. (**E**) Results for DDX4 expression.

bioRxiv preprint doi: https://doi.org/10.1101/2024.05.31.596483; this version posted June 1, 2024. The copyright holder for this preprint (which was not certified by peer review) is the author/funder, who has granted bioRxiv a license to display the preprint in perpetuity. It is made available under aCC-BY 4.0 International license.

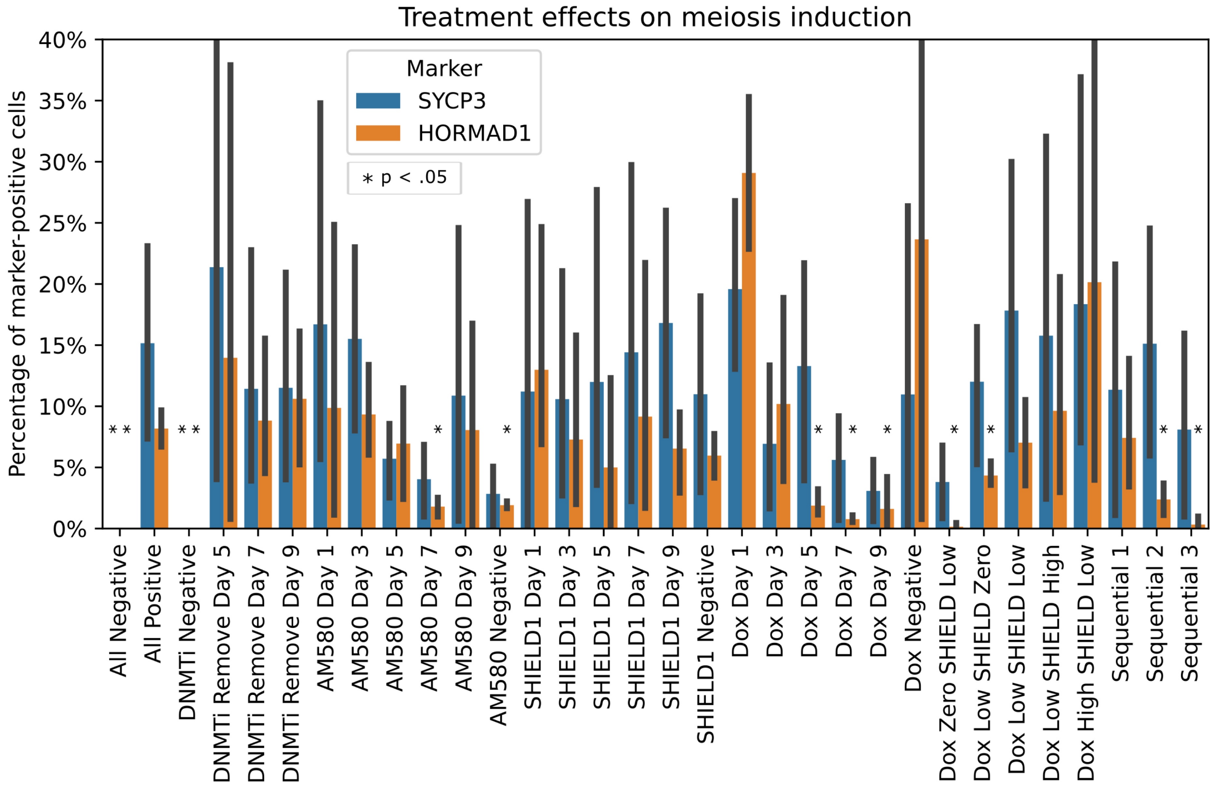

Fig. S6. Quantification of the effects of omitting various components of the meiosis induction protocol, or reducing their doses. Significance test comparisons are to the "all positive" control.

bioRxiv preprint doi: https://doi.org/10.1101/2024.05.31.596483; this version posted June 1, 2024. The copyright holder for this preprint (which was not certified by peer review) is the author/funder, who has granted bioRxiv a license to display the preprint in perpetuity. It is made available under aCC-BY 4.0 International license.

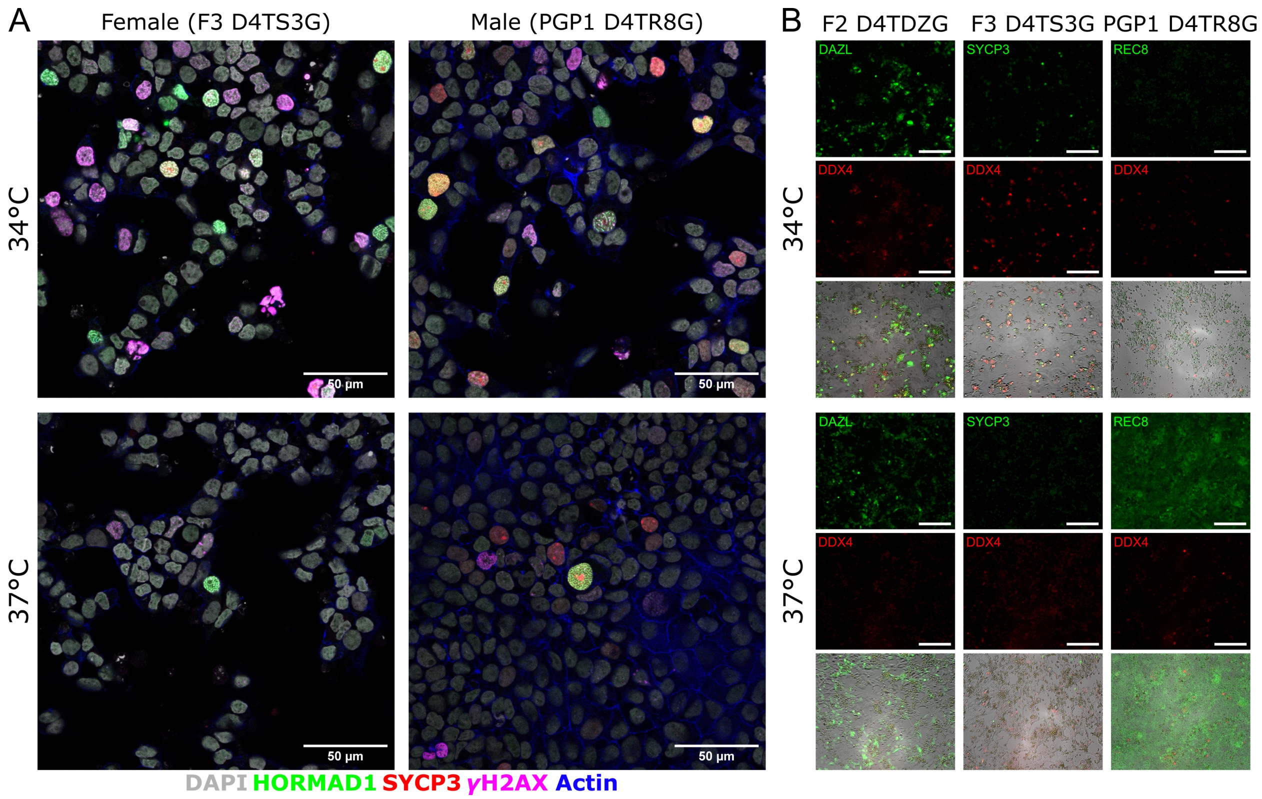

Fig. S7. Microscope images of meiosis induction from male and female hiPSCs at 34 °C vs. 37 °C. (**A**) Immunofluorescence microscopy, staining for DNA (DAPI; gray), HORMAD1 (green), SYCP3 (red), γH2AX (magenta), and actin (phalloidin; blue). Scale bar is 50 µm. (**B**) Live imaging of fluorescent reporter expression of DAZL, SYCP3, REC8, and DDX4. Scale bar is 200 µm.

bioRxiv preprint doi: https://doi.org/10.1101/2024.05.31.596483; this version posted June 1, 2024. The copyright holder for this preprint (which was not certified by peer review) is the author/funder, who has granted bioRxiv a license to display the preprint in perpetuity. It is made available under aCC-BY 4.0 International license.

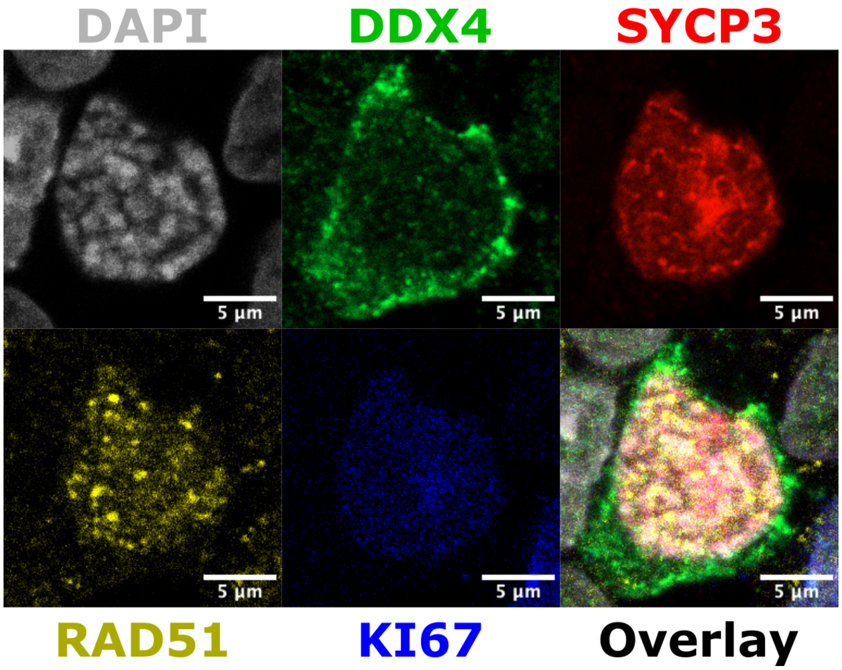

Fig. S8. Immuno-staining for DDX4, SYCP3, RAD51, and KI67 in a day 15 female meiotic cell. Cytoplasmic DDX4 staining, filamentous SYCP3 staining, and nuclear RAD51 foci are observed.

bioRxiv preprint doi: https://doi.org/10.1101/2024.05.31.596483; this version posted June 1, 2024. The copyright holder for this preprint (which was not certified by peer review) is the author/funder, who has granted bioRxiv a license to display the preprint in perpetuity. It is made available under aCC-BY 4.0 International license.

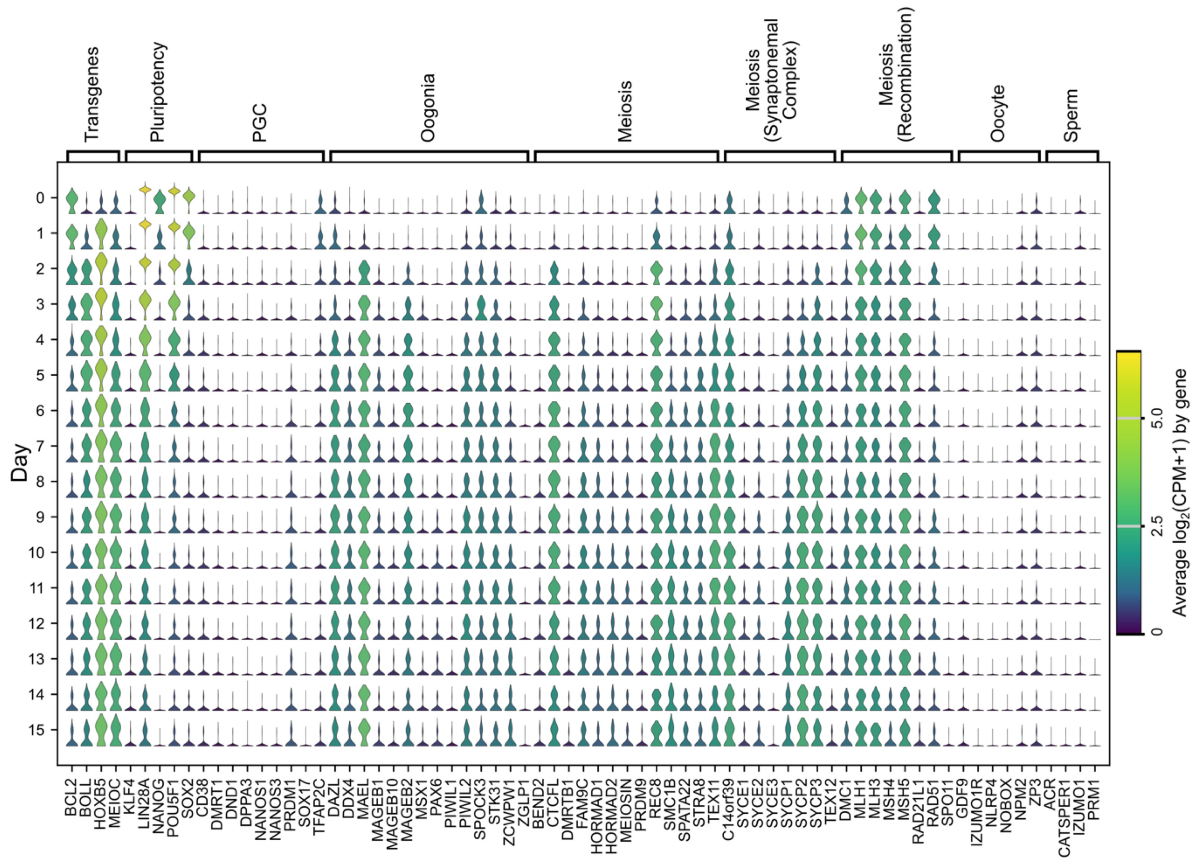

Fig. S9. Violin plots showing expression of marker genes from days 0 to 15 of meiosis induction. Units for the color scale are log_2(CPM+1). Categories include: exogenous transgenes, pluripotency markers, primordial germ cell markers, oogonia markers, meiosis markers, synaptonemal complex components, recombination markers, oocyte markers, and sperm markers.

bioRxiv preprint doi: https://doi.org/10.1101/2024.05.31.596483; this version posted June 1, 2024. The copyright holder for this preprint (which was not certified by peer review) is the author/funder, who has granted bioRxiv a license to display the preprint in perpetuity. It is made available under aCC-BY 4.0 International license.

Study	Summary of method	Outcome
Kee *et al*. 2009	Differentiation of hESCs in BMP-containing medium for 7 days, with overexpression of DAZL and BOLL.	Weak expression of SYCP3 by immuno-staining; REC8 and HORMAD1 expression not observed.
Medrano *et al*. 2011	Differentiation of hESCs by FGF withdrawal from culture medium for 14 days, with overexpression of DAZL and DDX4.	Cells differentiated without upregulating SYCP3 or REC8.
Eguizabal *et al*. 2011	Differentiation of hESCs by FGF withdrawal from culture medium for 21 days, followed by retinoic acid treatment for 21 days, followed by harvesting cells and re-plating in medium with forskolin, LIF, FGF, and CYP26 inhibitor for 14-28 days.	Cells overgrew during the initial 21-day differentiation. When the protocol was modified to start at the retinoic acid step, REC8 was upregulated but other markers were not expressed.
Easley *et al*. 2012	Culturing male hiPSCs in mouse spermatogonial stem cell maintenance medium for 10 days.	Cells differentiated without upregulating SYCP3 or REC8.
Jung *et al*. 2017	Treatment of hESCs with BMP4 for 1 hour, followed by overexpression of DAZL and BOLL for 7 days.	Weak expression of SYCP3 by immuno-staining; REC8 and HORMAD1 expression not observed.

Supplementary Table 1. Replication attempts of previous reported methods for inducing meiosis from human pluripotent stem cells. The methods were carried out as described in their respective papers (see References), although PiggyBac transposon plasmids were used instead of lentivirus for transgene overexpression.

bioRxiv preprint doi: https://doi.org/10.1101/2024.05.31.596483; this version posted June 1, 2024. The copyright holder for this preprint (which was not certified by peer review) is the author/funder, who has granted bioRxiv a license to display the preprint in perpetuity. It is made available under aCC-BY 4.0 International license.

id	Factor	Reason for inclusion	Barcodes
1	MEK1 S218D,S222D	CPO library	TGACATTAAGCAATTGGTGA
2	myr-AKT1	CPO library	GACTGTCTCCTTGTTAAAAT
3	RHEB Q64L	CPO library	CTGTTTGGCTCCATTGGCCA
4	IKKa S176E,S180E	CPO library	GAACGCGTTAAATTATTGCA
5	IKKb S177E,S181E	CPO library	GAGTCCGCACCAGGGCCGGC
6	STAT3 A662C,N664C,V667L	CPO library	TTCATATCGATCTTTAGCGC
7	CTNNB1 S33A,S37A,T41A,S45A	CPO library	AAATATTTATGACACCTGTA
8	GSK3beta K85A	CPO library	TCCGCTGATCGCAGTTCCCT
9	CTNNB1 S33Y	CPO library	GAGACATGCTGCTAAGCGGT
10	MAPK9	CPO library	ATATCCGTCGCAGAACAGTT
11	Mkk7-JNK2 fusion	CPO library	TTAAAAACAATCAGTAAAGA
12	MEK5 DDS311D,T315D	CPO library	ATCGTAGCTGTCGTTGTTCG
13	MEK5	CPO library	TGATGAGACTCTCATCATCA
14	Notch1 ICD	CPO library	GTAGTCACTTTTCCTTTTTG
15	Notch3 ICD	CPO library	TTATCATGTGCGTCCAAGAA
16	MAPK14	CPO library	GAATTTGATCGTGGTATTA
17	MKK6 S207E,T211E	CPO library	TTCTCTAGAGTATTGTTGAA
18	Gli truncation	CPO library	CTGTAAATTGTGTTGTGCCG
19	SMOM2 W535L	CPO library	GAAATATTTTCCTTGTTCCA
20	TGFbetaR1 T204D	CPO library	TCAAACGATCAAGTGAATGA
21	BCL2	CPO library	TTAATATCTGCTAAAAAAG
22	BCL-XL	CPO library	TCGAGTGATTGTACGCGATA
23	Caspase-8 C360A	CPO library	AAACAACAACGATCGAGTTA
24	Caspase-3 C163A	CPO library	GTTATTCCAAAGTTACAAGT
25	ERalpha Y537S	CPO library	GGGAGCTTTGGTAATCAAAC
26	AR-V7	CPO library	TAGACCCTACGGTCAAACTA
27	YAP2-8SA	CPO library	GGCAAGGCTTACGACAGTGA
28	EZH2	Regulon analysis (oogonia_meiotic)	CCATGTGACAAATTCCCATC
29	KCTD19	Known from literature	AGAAGACCTTGATAAGCGCT
30	BRCA1	Regulon analysis (oogonia_meiotic)	CAAGTTTGACAGACCTTCGA
31	DMRTB1	GRN analysis	TTGTAAGGCACACCGAGTCT
32	HMGB2	GRN analysis	GAACGGTCAACAACATTTAA
33	RAN	GRN analysis	GGCAGTGGTGAACGCAGATA
34	HMGB1	Regulon analysis (oogonia_STRA8)	CGAACAGTACACATCACGTC
35	HESX1	Regulon analysis (oogonia_meiotic)	TACCGAATGTAATGCCGCAC

bioRxiv preprint doi: https://doi.org/10.1101/2024.05.31.596483; this version posted June 1, 2024. The copyright holder for this preprint (which was not certified by peer review) is the author/funder, who has granted bioRxiv a license to display the preprint in perpetuity. It is made available under aCC-BY 4.0 International license.

36	RNF138	GRN analysis	TACAAAAAAATCATTTTTGA
37	HOXB5	Regulon analysis (oogonia_STRA8)	GATGAGCGCTGCATTTTTAC
38	HOXA5	Regulon analysis (oogonia_STRA8)	TTTTCTGGGGTGGCTCCCAA
39	HOXA10	Regulon analysis (oogonia_STRA8)	TTTATCGTTTGTACAATGAA
40	HMGB3	Regulon analysis (oogonia_STRA8)	GCCGGTAACGTCGGTGACGA
41	DMC1	GRN analysis	TGACGTTGGTTTGATAGCG
42	TERF1	GRN analysis	AGCAGGTTTATCTGATGCAG
43	ZCWPW1	Known from literature	TGACTAAGTATTCCATTGGC
44	RBM46	Known from literature	AATGACTTTGAGCCAACAGA
45	RFX5	Regulon analysis (oogonia_STRA8)	AAGAGGCACTTGCACTGTCA
46	CTCFL	Known from literature	TCTTTGTCGTATGGTGTCAA
47	MYBL1	Regulon analysis (oogonia_meiotic)	TTTGTATATTCCGGCAATGT
48	DMRTC2	GRN analysis	TTATGACTTTGACTTGACAA
49	ANHX	GRN analysis	GGCAACGCACTTGACGCCAG
50	RB1	Regulon analysis (oogonia_meiotic)	ACCTTACTTAAAATCGTTTC
51	MEIOSIN	Known from literature	CACACTCATTGAAACTTGA
52	SAP30	Regulon analysis (oogonia_meiotic)	TAAATGTGTGGGTAAAAGTT
53	HDAC2	Regulon analysis (oogonia_meiotic)	ATGCAATGCTAAGTTGGAGA
54	SMC3	GRN analysis	TGCTTGTGAGTTTGGTGGAT
55	KDM5B	Regulon analysis (oogonia_meiotic)	ACTGCGCTGTCTTTTTCAAA
56	SYMPK	Known from literature	GGTGCAGAAGCTAACTTTCC
57	MSX1	Regulon analysis (oogonia_STRA8)	CATATTTAGTTCTCCTTCTT
58	YTHDC2	Known from literature	TGCCTATTATAGGGGTGCCG
59	NELFE	Regulon analysis (oogonia_STRA8)	TGAGCAATATACAAAAGACA
60	ZNF541	Known from literature	CTTCATAAACCCATCGTTGA
61	NFYB	Regulon analysis (oogonia_meiotic)	GACTGTAACTGCGATTGCAA
62	MEIOC	Known from literature	GATTTTGAGTACGATTACTC
63	ESX1	GRN analysis	GAACCAGAAACAAAATAAAA
64	STRA8	Known from literature	GGGCAGGGTAACGAGGGGGG
65	PRDM9	GRN analysis	CCAGGAACTATCTATTCTTC
66	HDAC6	Regulon analysis (oogonia_meiotic)	GCCTTCCCTTCCAGATCCTA
67	RAD51	GRN analysis	AAGACCATCTGACTCAAACA
68	SOD1	GRN analysis	ATTGGCGGTGCGCTCGCCGA
69	UBB	GRN analysis	TGAATCACAGAAGAATACTC

bioRxiv preprint doi: https://doi.org/10.1101/2024.05.31.596483; this version posted June 1, 2024. The copyright holder for this preprint (which was not certified by peer review) is the author/funder, who has granted bioRxiv a license to display the preprint in perpetuity. It is made available under aCC-BY 4.0 International license.

70	RARG	Regulon analysis (oogonia_STRA8)	TAAGGAAGTGAGCCAGATCT
71	NFYC	Regulon analysis (oogonia_meiotic)	ATATTGCATGAAACCCTGTT;CACCGTTTCCCTGACTAAAA;GCTGCAATCTTGTAAGACCA
72	NOBOX	GRN analysis	AAATTCAAAGAAGATAAAAG;AAGATGTTAGAATCAAGAAG;GCCAACTACAATAAATATTC
73	FIGLA	GRN analysis	ATTGACTATGACACCGATGA;TTATTGGCGAGGTTAACAAC;GTAAATGTCCTTAATAACGT
74	DAZL	Known from literature	ACAGCGAACGAACATGATCT;GATATATTTAGTTGCTTCGC;AGTTTGTCTGTAAGCGAAAC
75	BOLL	GRN analysis and known from literature	ATTGGGGGTAAGTCTATCTT;ATGATCATTGTATATACCGT;ATGGGGCTCGGAGCAGGACA
76	DDX4	GRN analysis and known from literature	CATAAGCTTCCTTGTCCTAC;GGTGCAGGAGCTAACTTTCC;TAAAAACCGGGCTTTTTCAC
77	ELK1	Regulon analysis (oogonia_STRA8)	GCAGGAAGCGTTGTGTTGCA;ATCCCTTCTCACCAACCACC
78	TAF4B	Known from literature	ACTAACAATCCAGAACTTGC;TTCTTCAATGCTGGGTGATA
79	DNMT1 CRISPRi	DNA demethylation	ATAGAGAGACCATTAATGGGTCTCCTAGG
80	DPPA3	DNA demethylation	GATAGCATGACTTATGAAAT
81	TET1	DNA demethylation	GGTATTTTGCCCGTGATTCT
82	TET3	DNA demethylation	AGATCTCTAGAAATCTAAAA
83	SMAD1-active	BMP signaling	AATACCTATCACTATAGCTT
84	SMAD9-active	BMP signaling	CACAGAGGTCGGACGAAGTT
85	DMRT1	Known from literature	TTACTTCGGAAACTGAAGAG;TTATTGGAGACAACAACGCA
86	PAX5	Known from literature	GCTGCCTCTCAGATTTCTGA
87	PAX6 isoform a	GRN analysis	CTTTTCAAATAAGACGTCTA
88	PAX6 isoform b	GRN analysis	ATTGAAGGACTTTTCAGTTA

Supplementary Table 2. Factors screened for meiosis induction. #1-78 were included in barcode enrichment screening, and #79-88 were added for scRNAseq screening.

bioRxiv preprint doi: https://doi.org/10.1101/2024.05.31.596483; this version posted June 1, 2024. The copyright holder for this preprint (which was not certified by peer review) is the author/funder, who has granted bioRxiv a license to display the preprint in perpetuity. It is made available under aCC-BY 4.0 International license.

DMC1
HORMAD1
HORMAD2
INCA1
MEIOB
PRDM9
RAD51AP2
SCML1
SHCBP1L
SMC1B
SPATA22
SPDYA
SPO11
SYCE2
SYCE3
SYCP1
SYCP2
SYCP3
TEX12

Supplementary Table 3. Genes used for meiosis gene score calculation.

bioRxiv preprint doi: https://doi.org/10.1101/2024.05.31.596483; this version posted June 1, 2024. The copyright holder for this preprint (which was not certified by peer review) is the author/funder, who has granted bioRxiv a license to display the preprint in perpetuity. It is made available under aCC-BY 4.0 International license.

Name	Sequence	Purpose
MPS542_Barcode pulldown_3'_F	/5BiosG/GTTTTAGAGCTAGAAATAGC	Barcode capture (scRNAseq)
MPS543_Barcode pulldown_3'_R	/5BiosG/TCCAAACTCATCAATGTATC	Barcode capture (scRNAseq)
MPS546_Illumina_p5	AATGATACGGCGACCACCGAGATCT	Barcode capture (scRNAseq)
MPS547_Illumina_p7	CAAGCAGAAGACGGCATACGAGAT	Barcode capture (scRNAseq)
oAMP123_barcode_F	ACACTCTTTCCCTACACGACGCTCTTCCGATCTNNATACTCAGAAGATGTCACTCACC	Barcode enrichment
oAMP124_barcode_R	GAGTTCAGACGTGTGCTCTTCCGATCTNNNCATTCATAGTTCTTGCTCAGTGG	Barcode enrichment
MPS017_REC8_5'arm_fwd	TGGAGCCCAGTGTTCCCCTTATG	Cloning
MPS018_REC8_seq_fwd	CAAGAAAAGCCATATGGTCGC	Cloning
MPS019_REC8_5'_rev	GTGGAATCTGGGCCCCGGCT	Cloning
MPS020_REC8_3'fwd	GGTTAGAGTCCATTTACAAAGCTGC	Cloning
MPS021_REC8_rev	TAGAAGTGGCCGGTTTCCTG	Cloning
MPS022_REC8_3'rev	TGCTCTCTGAAGAGATTTTGCC	Cloning
MPS458_BB_SYCP3 Gibson	caaataggggttccgcgcacatttccccgTCCGAGACCTAAATTGCGGG	Cloning
MPS459_SYCP3_mGL Gibson	GTTTTTTAAAGTCAGGAAGCATCTAAATATGGTGTCCaagggcgaggagctgttcac	Cloning
MPS460_SYCP3_5'_rev	GGACACCATATTTAGATGCT	Cloning
MPS461_T2A-SYCP3-Gibson	CCTGGAATACTTTTTTCCGGAGGACACCATtgggccaggattctcctcga	Cloning
MPS462_SYCP3_3'_fwd	ATGGTGTCCTCCGGAAAAAAG	Cloning
MPS463_SYCP3_BB Gibson	GAAAACCACACTGACAAGATCTGGAGGTTCTTCTATGTGAGAACAAGGCAT	Cloning
MPS166_REC8_1F_CRISPRa	caccgCCTGGCAACAGGGTCTCCCG	CRISPRa sgRNA
MPS167_REC8_1R_CRISPRa	aaacCGGGAGACCCTGTTGCCAGGc	CRISPRa sgRNA
MPS168_REC8_2F_CRISPRa	caccgTGACAGCCAATGGGGAACGG	CRISPRa sgRNA
MPS169_REC8_2R_CRISPRa	aaacCCGTTCCCCATTGGCTGTCAc	CRISPRa sgRNA
MPS170_REC8_3F_CRISPRa	caccGTCGGGAACGCCAAGTATCC	CRISPRa sgRNA
MPS171_REC8_3R_CRISPRa	aaacGGATACTTGGCGTTCCCGAC	CRISPRa sgRNA
MPS172_REC8_4F_CRISPRa	caccgCCTCGGGAGACCCTGTTGCC	CRISPRa sgRNA
MPS173_REC8_4R_CRISPRa	aaacGGCAACAGGGTCTCCCGAGGc	CRISPRa sgRNA
MPS479_SYCP3_F_CRISPRa	accGCGCCCAAATAGCTGGCCCA	CRISPRa sgRNA
MPS480_SYCP3_R_CRISPRa	aacTGGGCCAGCTATTTGGGCGC	CRISPRa sgRNA
MPS023_REC8_outer_5'	ACTTCTCCCATCCCCAGGTC	Genotyping
MPS024_REC8_outer_3'	GCCACCACACCATACATTTCAATC	Genotyping
MPS489_SYCP3_outer_5'	TGTCGAAATCTTCTGCCTGTGT	Genotyping
MPS490_SYCP3_outer_3'	ACCATTCACTCTTAACATCATGGA	Genotyping
MPS064_REC8_cterm sgRNA_1 FWD	caccGCCGGGGCCCAGATTCCACTG	Knock-in sgRNA
MPS065_REC8_cterm sgRNA_1 REV	aaacCAGTGGAATCTGGGCCCCGGC	Knock-in sgRNA
MPS066_REC8_cterm sgRNA_2 FWD	caccGTAAATGGACTCTAACCTCAG	Knock-in sgRNA
MPS067_REC8_cterm sgRNA_2 REV	aaacCTGAGGTTAGAGTCCATTTAC	Knock-in sgRNA
MPS068_REC8_cterm sgRNA_4 REV	aaacGGGCCCAGATTCCACTGAGGC	Knock-in sgRNA
MPS069_REC8_cterm sgRNA_4 FWD	caccGCCTCAGTGGAATCTGGGCCC	Knock-in sgRNA
MPS070_REC8_cterm sgRNA_5 REV	aaacGATTCCACTGAGGTTAGAGTC	Knock-in sgRNA
MPS071_REC8_cterm sgRNA_5 FWD	caccGACTCTAACCTCAGTGGAATC	Knock-in sgRNA
MPS072_REC8_cterm sgRNA_3 REV	aaacAGATTCCACTGAGGTTAGAGC	Knock-in sgRNA
MPS073_REC8_cterm sgRNA_3 FWD	caccGCTCTAACCTCAGTGGAATCT	Knock-in sgRNA
MPS464_SYCP3_sgRNA_fwd	caccGCATCTAAATATGGTGTCCTC	Knock-in sgRNA
MPS465_SYCP3_sgRNA_rev	aaacGAGGACACCATATTTAGATGC	Knock-in sgRNA
qMPS001F_GAPDH	GGTGACCAGGCGCCCAATACGA	qPCR
qMPS001R_GAPDH	CGCTTCGCTCTCTGCTCCTCCTGT	qPCR
qMPS070F_DPPA3	ACGCCGATGGACCCATCACAGTTT	qPCR
qMPS070R_DPPA3	TCTCGGAGGAGATTTGAGAGGCCC	qPCR
qMPS071F_REC8	TACCTGCTCCTGGTGCTCTC	qPCR
qMPS071R_REC8	TGGATCAGGAGGCGACCATA	qPCR
qMPS072F_SYCP3	TCTACTTACTGGTGCACAAAATGA	qPCR
qMPS072R_SYCP3	TCTCTTGCTGCTGAGTTTCCA	qPCR

bioRxiv preprint doi: https://doi.org/10.1101/2024.05.31.596483; this version posted June 1, 2024. The copyright holder for this preprint (which was not certified by peer review) is the author/funder, who has granted bioRxiv a license to display the preprint in perpetuity. It is made available under aCC-BY 4.0 International license.

qMPS073F_MAX	GGACTCGGCTTGTTGTTGTC	qPCR
qMPS073R_MAX	GGTTGCTCTTCGTCGCTCT	qPCR
qMPS074F_MGA	ACCGAACAGAATAACCCGCC	qPCR
qMPS074R_MGA	AATGGCCTTCCCATCCGTG	qPCR
qMPS075F_E2F6	ACCCAGTCTCCTCCTGGAC	qPCR
qMPS075R_E2F6	TATTTTTGATGGCAGCAGGC	qPCR
qMPS076F_RNF2	GCAGGAGCCGCAATGTCT	qPCR
qMPS076R_RNF2	ATTGCCTCCTGAGGTGTTCG	qPCR
qMPS077F_PCGF6	GAGGACGAGGACGAGGAGTT	qPCR
qMPS077R_PCGF6	GATTAATCAGGCGCTCCTCC	qPCR
qMPS078F_DNMT1	CCCCAAAGAACCAACGGAGA	qPCR
qMPS078R_DNMT1	CTGAATGCACTTGGGAGGGT	qPCR
qMPS079F_DNMT3A	CACCGGCCATACGGTGGAG	qPCR
qMPS079R_DNMT3A	TGTTGAGCCCTCTGGTGAAC	qPCR
qMPS080F_DNMT3B	GGAGATTCGCGAGCCCAG	qPCR
qMPS080R_DNMT3B	CTCCCTTCATGCTTTCCTGC	qPCR
qMPS081F_UHRF1	ACAGGGGCAAACAGATGGAG	qPCR
qMPS081R_UHRF1	TGGATGGTGTCATTCAGGCG	qPCR
qMPS082F_SETDB1	GTTGTGAGTCTGGGGTCTGG	qPCR
qMPS082R_SETDB1	ATGCTTTTGTCCTCTCCCGT	qPCR

Supplementary Table 4. Oligos used in this study.

bioRxiv preprint doi: https://doi.org/10.1101/2024.05.31.596483; this version posted June 1, 2024. The copyright holder for this preprint (which was not certified by peer review) is the author/funder, who has granted bioRxiv a license to display the preprint in perpetuity. It is made available under aCC-BY 4.0 International license.

**Primary Antibodies**					
**Target**	**Antibody type**	**Supplier**	**Catalog#**	**Dilution**	**RRID**
SYCP3	Rabbit IgG, polyclonal	Abcam	ab15093	1:250	AB_301639
DDX4	Rabbit IgG, polyclonal	Abcam	ab13840	1:250	AB_443012
KI67	Rat IgG, monoclonal	Thermo	14-5698-37	1:250	AB_2865119
HORMAD1	Rabbit IgG, polyclonal	Proteintech	13917-1-AP	1:250	AB_2120844
γH2AX	Mouse IgG, monoclonal	Millipore Sigma	05-636	1:500	AB_309864
Centromere	Human IgG, polyclonal, FITC-labeled	Antibodies Inc	15-235-F	1:200	AB_2797147
SYCP3	Goat IgG, polyclonal	R&D Systems	AF3750	1:250	AB_2197194
T2A	Rat IgG, monoclonal	Millipore Sigma	MABE1923	1:250	AB_3097817
TEX12	Rabbit IgG, polyclonal	Abcam	ab122455	1:250	AB_11128111
RAD51	Mouse IgG, monoclonal	Novus	NB100-148	1:250	AB_10002131
**Secondary antibodies (all donkey polyclonal)**					
**Target**	**Fluorophore**	**Supplier**	**Supplier Number**	**Dilution**	**RRID**
Goat IgG	AF568	Thermo Fisher	A11057	1:500	AB_2534104
Mouse IgG	AF647	Thermo Fisher	A31571	1:500	AB_162542
Rabbit IgG	AF488	Jackson	711-545-152	1:500	AB_2313584
Rat IgG	CF568	Sigma	SAB4600077	1:500	AB_2827516
Rabbit IgG	AF568	Thermo Fisher	A10042	1:500	AB_2534017
Rat IgG	Dylight755	Thermo Fisher	SA5-10031	3:500	AB_2556611

Supplementary Table 5. Antibodies used in this study.

bioRxiv preprint doi: https://doi.org/10.1101/2024.05.31.596483; this version posted June 1, 2024. The copyright holder for this preprint (which was not certified by peer review) is the author/funder, who has granted bioRxiv a license to display the preprint in perpetuity. It is made available under aCC-BY 4.0 International license.

#	Cell line	Factors	Sorting	Total Cells	Germ cell	Germ cell mitotic	PGC	Oogonia STRA8	Oogonia meiotic	Pre-oocyte	Pre-spermat ogonia
1	PGP1 D4TR8G	BCL2, HOXB5, and STRA8	unsorted	3553	1323	350	257	1605	0	0	18
2	F3 D4TS3G	BCL2, HOXB5, and STRA8	unsorted	5308	1187	320	299	3464	4	0	34
3	PGP1 D4TR8G	BCL2, HOXB5, STRA8, and the pool of 88 factors	unsorted	90809	32279	10728	9246	37839	27	1	689
4	PGP1 D4TR8G	BCL2, HOXB5, STRA8, and the pool of 88 factors	sorted DDX4+	59668	20629	6630	6399	24732	65	6	1207
5	PGP1 D4TR8G	BCL2, HOXB5, STRA8, and the pool of 88 factors	sorted REC8+	76522	26398	11405	9701	28595	14	0	409
6	F3 D4TS3G	BCL2, HOXB5, STRA8, and the pool of 88 factors	unsorted	50837	14834	4481	3267	27916	38	1	300
7	F3 D4TS3G	BCL2, HOXB5, STRA8, and the pool of 88 factors	sorted DDX4+	59742	14949	6030	5233	32593	100	4	833
8	F3 D4TS3G	BCL2, HOXB5, STRA8, and the pool of 88 factors	sorted SYCP3+	88342	23339	6991	5549	51541	238	6	678
9	PGP1 D4TR8G	Pool of 88 factors	unsorted	10069	3607	2197	2213	2019	1	0	32
10	PGP1 D4TR8G	Pool of 88 factors	sorted DDX4+	28038	9798	6119	5401	6617	2	0	101
11	PGP1 D4TR8G	Pool of 88 factors	sorted REC8+	27982	10776	5316	5497	6114	6	1	272
12	F3 D4TS3G	Pool of 88 factors	unsorted	35747	9359	9648	9883	6772	1	0	84
13	F3 D4TS3G	Pool of 88 factors	sorted DDX4+	25380	10364	4287	4396	5996	11	1	325
14	F3 D4TS3G	Pool of 88 factors	sorted SYCP3+	32779	12425	5816	5202	8966	34	0	336
15	Mix of PGP1 D4TR8G and F3 D4TS3G	No factors	unsorted	5312	1227	1880	2016	189	0	0	0

Supplementary Table 6. List of samples in the scRNAseq screening experiment, and numbers of annotated cell types.
